# Dialing in single-site reactivity of a supported calixarene-protected tetrairidium cluster catalyst†
†Electronic supplementary information (ESI) available: Detailed characterization of Ir_4_ clusters, raw kinetic data, time scale analysis, experimental methods, and sample preparation. See DOI: 10.1039/c7sc00686a



**DOI:** 10.1039/c7sc00686a

**Published:** 2017-05-04

**Authors:** Andrew Palermo, Andrew Solovyov, Daniel Ertler, Alexander Okrut, Bruce C. Gates, Alexander Katz

**Affiliations:** a Department of Chemical Engineering , University of California at Davis , One Shields Avenue , Davis , California 95616 , USA . Email: bcgates@ucdavis.edu; b Department of Chemical and Biomolecular Engineering , University of California at Berkeley , Berkeley , California 94720-1462 , USA . Email: alexander.okrut@berkeley.edu ; Email: askatz@berkeley.edu

## Abstract

A closed Ir_4_ carbonyl cluster, **1**, comprising a tetrahedral metal frame and three sterically bulky *tert*-butyl-calix[4]arene(OPr)_3_(OCH_2_PPh_2_) (Ph = phenyl; Pr = propyl) ligands at the basal plane, was characterized with variable-temperature ^13^C NMR spectroscopy, which show the absence of scrambling of the CO ligands at temperatures up to 313 K. This demonstration of distinct sites for the CO ligands was found to extend to the reactivity and catalytic properties, as shown by selective decarbonylation in a reaction with trimethylamine N-oxide (TMAO) as an oxidant, which, reacting in the presence of ethylene, leads to the selective bonding of an ethyl ligand at the apical Ir site. These clusters were supported intact on porous silica and found to catalyze ethylene hydrogenation, and a comparison of the kinetics of the single-hydrogenation reaction and steady-state hydrogenation catalysis demonstrates a unique single-site catalyst—with each site having the same catalytic activity. Reaction orders in the catalytic ethylene hydrogenation reaction of approximately 1/2 and 0 for H_2_ and C_2_H_4_, respectively, nearly match those for conventional noble-metal catalysts. In contrast to oxidative decarbonylation, thermal desorption of CO from silica-supported cluster **1** occurred exclusively at the basal plane, giving rise to sites that do not react with ethylene and are catalytically inactive for ethylene hydrogenation. The evidence of distinctive sites on the cluster catalyst leads to a model that links to hydrogen-transfer catalysis on metals—involving some surface sites that bond to both hydrocarbon and hydrogen and are catalytically engaged (so-called “*” sites) and others, at the basal plane, which bond hydrogen and CO but not hydrocarbon and are reservoir sites (so-called “S” sites).

## Abstract 2

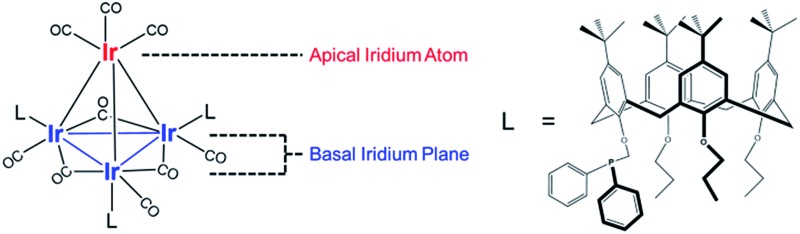
A closed Ir_4_ carbonyl cluster, **1**, comprising a tetrahedral metal frame and three *tert*-butyl-calix[4]arene(OPr)_3_(OCH_2_PPh_2_) (Ph = phenyl; Pr = propyl) ligands at the basal plane, was silica supported and consists of “*” and “S” sites, which could be dialed in selectively for controlling ethylene hydrogenation catalysis.

## Introduction

The majority of industrial catalysts (∼80%) are solids,^1^ and almost all of them have been discovered empirically,^2^ one application at a time, rather than by rational design based on understanding of broad fundamental principles—such understanding is in general hindered by the complexity and heterogeneity of catalyst surfaces.^3–6^ The goal of design of catalysts for whole classes of reactions through understanding of relationships between molecular-level structure and reactivity of active sites is most readily realized when the catalysts are molecular.^7–11^ In the work reported here, we investigated supported molecular catalysts with the goal of developing an approach to dial in those sites that are catalytically active—and not those that are inactive. All of these sites are part of isolated supported clusters, each of which consists of a ligated tetrahedron of Ir atoms dispersed on the surface of a high-area porous silica support and investigated with gas-phase reactants to prevent any complications of solvents. We use results from spectroscopic investigations and electronic structure calculations and a simple catalytic test reaction, ethylene hydrogenation, which involves readily identifiable reaction intermediates. Our demonstration of how to control the synthesis of catalytically active sites on a cluster that exhibits a heterogeneous population of sites has ramifications that extend to numerous technologically important reactions involving hydrogen transfer, such as ring opening and hydrodesulfurization of compounds in petroleum to manufacture clean-burning fuels, and hydrodeoxygenation of compounds derived from biomass to manufacture chemicals and fuels.^12–26^ Reactions in this class include alkene hydrogenation and alkane hydrogenolysis catalyzed by noble metals. Landmark publications addressing these reactions demonstrate two separate and distinct catalytic sites on metal surfaces.^27–38^ On some of them (denoted “*”), hydrogen and hydrocarbon reactants bond competitively and react, whereas on others (denoted “S”),^33^ hydrocarbons do not bond, and hydrogen bonds unproductively, reacting only after it migrates to nearby “*” sites.^27–31^

Extensive research has not yet led to a physical model of the “*” and “S” sites or answers to the question of how they discriminate between hydrogen and hydrocarbons.^33^ Most hypotheses about these sites have focused on sterics, with “S” sites suggested to lie between surface hydrocarbon species (*e.g.*, dehydrogenated carbonaceous deposits) and too tightly confined for hydrocarbon adsorption.^29,36–39^ Because researchers have been unable to identify these sites, they have not been able to control them. We now report elucidation of distinctive metal sites on the simplest polyhedron, the tetrahedral frame of Ir_4_ clusters—and how to select them for control of catalytic properties. We stress, on the one hand, the contrast between these molecular metal clusters and larger nanoparticles/extended metal surfaces (with multiple facets) as multisite platforms for reaction and catalysis,^40,41^ and, on the other hand, the contrast between the clusters, nanoparticles, and surfaces (with neighboring metal sites) and almost all of the supported single-site catalysts that have drawn recent attention,^3,5,29–31,42–46^ which consist of single, isolated metal sites on oxide surfaces.

**Fig. 1:**
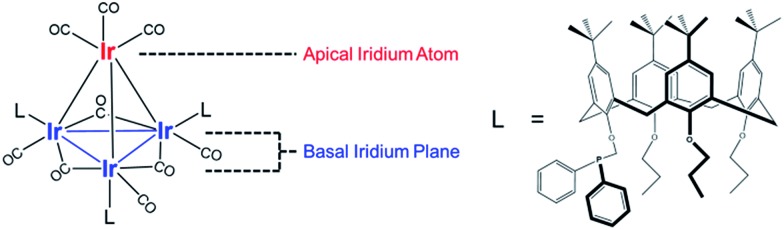
Schematic representation of a trisubstituted tetrairidium carbonyl cluster, closed cluster **1** (Ir_4_(CO)_9_L_3_), with three bulky phosphine ligands on the basal plane of the tetrahedron (left); structure of calix[4]-arene phosphine ligand L shown at right.

Our catalyst consists of silica-supported Ir_4_L_3_(CO)_9_, **1**, where L = *tert*-butyl-calix[4]arene(OPr)_3_(OCH_2_PPh_2_) (Pr = propyl; Ph = phenyl), and the bulky calixarene phosphine ligands are bonded exclusively to the basal plane of the tetrahedral cluster frame. Key advantages of the Ir_4_ cluster as a catalytic platform are its known structural stability and full characterization in the crystalline state (the structure is represented in Fig. 1),^47^ with IR and NMR spectroscopies providing essential details of its chemistry.

**Fig. 2:**
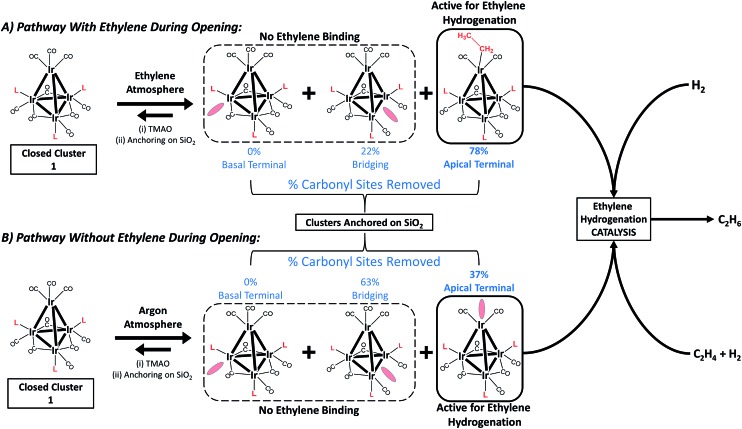
Schematic representation of reactive decarbonylation (vacant carbonyl sites represented by red ovals) of tetrairidium carbonyl cluster **1** (which incorporates terminal and bridging CO ligands, the latter in the basal plane) performed with (A) and, alternatively, without an ethylene atmosphere (B), leading to a single-site catalyst for ethylene hydrogenation (in red) at the apical position.

Fig. 2 summarizes our approach for opening sites occupied by CO on cluster **1** *via* reactive decarbonylation with trimethylamine N-oxide (TMAO) as the oxidant; we have previously shown that TMAO oxidizes CO ligands in **1** to synthesize sites that lead to ethylene bonding to the cluster (*vide infra*).^44^ On the basis of electronic structure calculations, which demonstrate a 8.5 kcal mol^–1^ preference in the electronic energy for bonding of ethylene to apical *versus* basal-plane Ir sites^44^—as a consequence of electronic rather than steric effects—we surmised that these sites were ones previously occupied by apical CO in **1**. These sites are contrasted with sites synthesized from **1** *via* thermal CO loss (simple desorption)—these latter sites bond to hydrogen and CO, but not ethylene, even when treated with ethylene over prolonged periods (*vide infra*).^44^ The synthesis of these latter sites occurs preferentially when dissociation of CO ligands from **1** is rate limiting, by an SN_1_ reaction mechanism. Such CO dissociation results in coordinative unsaturation selectively at phosphine-substituted Ir atoms located on the basal plane of the cluster, as previously explained by a *cis*-effect.^48–51^

Here, we show how the selectivity for the opening of basal-plane *versus* apical CO sites can be controlled by the simple presence of a reactive atmosphere of the ligand ethylene during decarbonylation in TMAO. Thus, when the oxidative decarbonylation was carried out as before, in the absence of ethylene, it took place primarily at basal-plane sites, as shown in Fig. 2B. However, when it was carried out instead in the presence of ethylene, the decarbonylation took place selectively at the apical sites, to give ethyl ligands bound there, as shown in Fig. 2A.

## Results

### Reactions of tetrairidium clusters and lack of fluxionality of 1 in liquid phase characterized by ^13^C NMR spectroscopy

The structure of **1** determined by single-crystal X-ray diffraction crystallography provides a starting point for understanding the reactivity and catalytic properties of substituted Ir_4_ clusters, and NMR spectroscopy is the essential technique that provides characterization of dynamic processes in the ligand sphere. A Ir_4_ carbonyl cluster substituted with a single PPh_2_Me ligand has been shown to be fluxional, undergoing three different exchange processes at low temperature (185 K).^52^ This fluxionality deterred previous investigators from assigning discrete sites for CO dissociation from the Ir_4_ frame.^50,52,53^ To the best of our knowledge, there are no data characterizing the fluxionality of trisubstituted Ir_4_ carbonyl clusters, such as **1**, besides a brief mention that such clusters are expected to have a higher activation barrier for CO exchange compared with monosubstituted ones.^52^ Thus, we used quantitative ^13^C NMR spectroscopy to characterize **1** in CDCl_3_, using an inverse gated decoupling (zgig) pulse program (*T*1 = 3 s, *d*1 = 15 s), with a total time for data acquisition per spectrum of 600 min, with data collected at room temperature and at 313 K. The detailed data characterizing all of the carbon resonances in **1** and their integration are included in Fig. S1–S13.† These data give no evidence of CO scrambling processes and therefore demonstrate discrete CO bonding sites in **1** that do not undergo exchange. This is a key result because it allows us to use spectroscopic methods and unequivocally link the static structure of crystalline **1** to that of the cluster in solution or on a partially dehydroxylated porous silica support.

### Reactions of tetrairidium clusters in liquid phase characterized by IR spectroscopy

In the following section, we first summarize results obtained by IR spectroscopy characterizing the activation of cluster **1** and its anchoring to the porous silica support. Data characterizing key reaction intermediates demonstrate our control of the ligand environment of the cluster and the influence of ethylene in steering this environment during oxidative removal of CO ligands. The results further demonstrate how to dial in the fraction of sites on the cluster that are active for ethylene hydrogenation catalysis—these sites are created when **1** is oxidatively decarbonylated by treatment with TMAO.

**Fig. 3:**
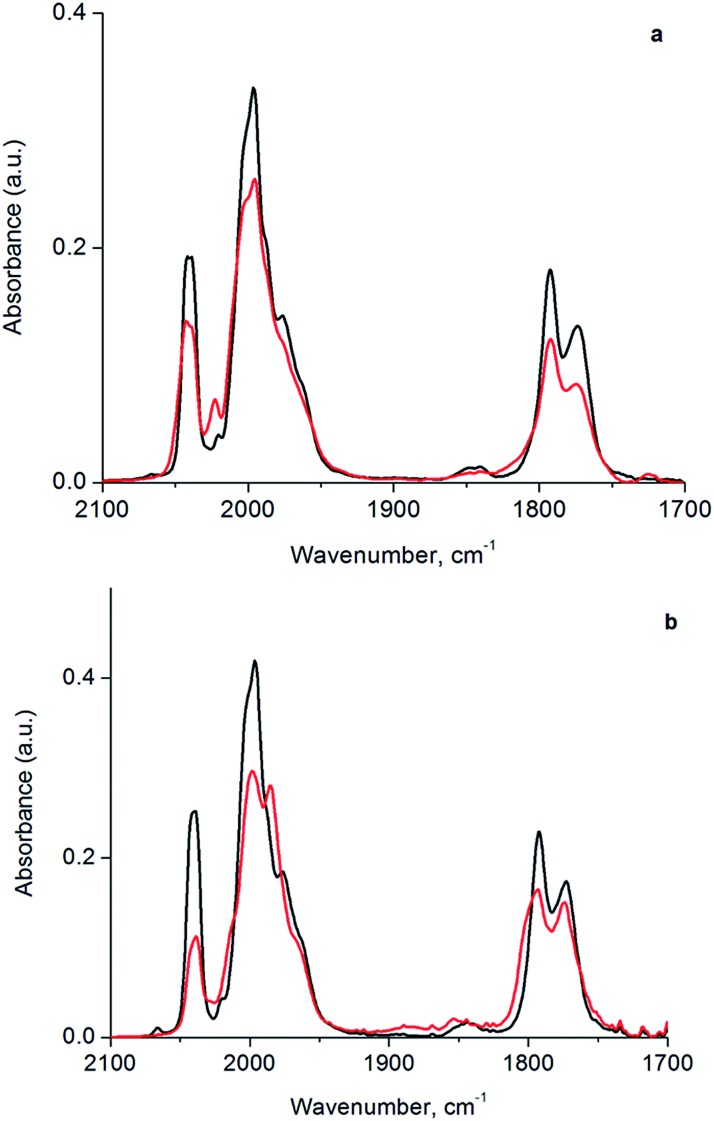
IR spectra characterizing anhydrous *n*-decane solutions of cluster **1** before (black line) and after (red line) opening with trimethylamine N-oxide (TMAO) in 1 bar of an (a) argon and (b) ethylene atmosphere.

To elucidate the role of ethylene during TMAO opening of sites, we performed an analysis of IR data characterizing reactive decarbonylation of cluster **1** with and without ethylene present, by measuring the relative peak areas in the IR spectra of terminal and bridging CO ligands before and after TMAO treatment, in *n*-decane solvent at room temperature. The data show that, irrespective of whether ethylene was present, TMAO treatment led to the removal of CO from the cluster in a manner that did not lead to changes in the CO band frequencies. The integrated areas (Fig. 3 and Table 1) demonstrate the removal of 1.3 CO ligands per cluster when TMAO treatment was conducted in the absence of ethylene and the removal of 1.4 CO ligands per cluster when it was conducted in the presence of ethylene. Because the IR spectra distinguish terminal and bridging CO ligands in the cluster (bands between 1950 and 2050 cm^–1^ represent terminal CO ligands, whereas those between 1750 and 1850 cm^–1^ represent bridging CO ligands), our analysis of the spectra indicates that, in the absence of ethylene, 0.48 of the original 6 terminal and 0.81 of the original 3 bridging CO ligands in **1** (Fig. 1) were removed per cluster, whereas, in the presence of ethylene, 1.1 terminal and 0.33 bridging CO ligands were removed. Thus, we conclude that in the absence of ethylene, 63% of the removed CO ligands originally occupied bridging positions and must therefore have been located in the basal plane.^44^

**Table 1:** Relative changes in IR band areas characterizing cluster **1** when decarbonylated with TMAO in an atmosphere of argon or an atmosphere of ethylene

Sample	Relative terminal carbonyl band area	Relative bridging carbonyl band area
Cluster **1**	1.0	1.0
Cluster **1** treated with TMAO in Ar	0.92	0.73
Cluster **1** treated with TMAO in C_2_H_4_	0.81	0.89

However, when oxidative decarbonylation by TMAO was performed in an ethylene atmosphere, only 22% of the removed CO ligands were bridging. This result means that ethylene selectively steers carbonyl removal during TMAO treatment among the three possible sites for reaction (Fig. 2): apical terminal, basal terminal, and bridging carbonyl sites. A more precise assignment of exactly which terminal sites are removed is not possible *via* IR spectroscopy, because each terminal IR band has multiple contributions, from both apical and basal-plane CO ligands, as shown previously by electronic structure calculations.^47^ Determination of which of the terminal CO ligands were removed from apical and which from basal-plane sites (Fig. 2) required additional experiments, involving analysis of catalytic reaction kinetics. The results of these experiments are presented below; they opened the way to a determination of how by choosing the method of CO removal from the cluster we could selectively direct the synthesis of sites from which CO removal occurred specifically to those that are active for ethylene hydrogenation catalysis.

### Reactivity of silica-supported tetrairidium clusters characterized by IR spectroscopy

The decarbonylated clusters were physisorbed onto partially dehydroxylated porous silica from a slurry in *n*-hexane, to synthesize catalysts containing 1.0 wt% Ir. Following removal of the *n*-hexane by evacuation, the solid samples were characterized by IR spectroscopy (Fig. S14 and Table S1†). The carbonyl bands characterizing the soluble and supported clusters were essentially the same in terms of frequencies and relative band areas for the dissolved and supported clusters (Fig. S14†), showing that adsorption on silica—known to be a weakly interacting support for Ir_4_ clusters^54^—led to no significant structural changes in the cluster frame or its ligands.

**Fig. 4:**
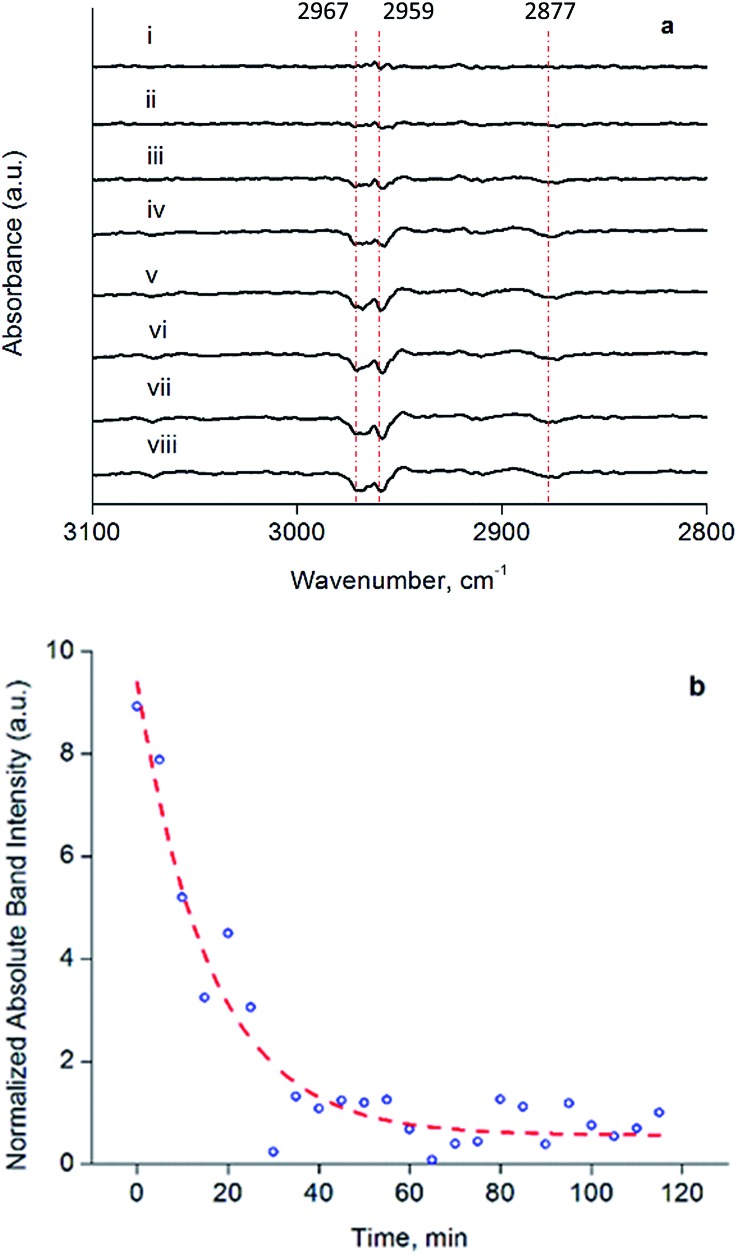
(a) Difference spectra in *ν*_CH_ region of silica-supported samples formed from **1** by reactive decarbonylation with TMAO in the presence of ethylene. Data represent changes occurring in a flow system under the following conditions: (i) helium flowing at a rate of 10 mL min^–1^ and subsequently H_2_ flowing at a rate of 10 mL min^–1^ of for the following times (min): (ii) 5, (iii) 10, (iv) 15, (v) 20, (vi) 30, (vii) 60, (viii) 120. Subtraction spectra are referenced to that of the original sample following TMAO treatment and anchoring on silica. (b) Change of 2959 cm^–1^ band characterizing loss of bound ethyl intermediate during H_2_ treatment. All spectra were recorded at 313 K and 1 bar.

To investigate the structure and reactivity of the supported clusters after oxidative decarbonylation with TMAO in the presence of ethylene, we used a difference IR technique because it enabled us to identify small changes in bound hydrocarbon intermediates,^55^ notwithstanding a background of high-intensity calixarene-phosphine bands. These intermediates became evident in the *ν*_CH_ stretching modes of Ir-bound ethyl as bands at 2967, 2959, and 2877 cm^–1^ in the difference IR spectra of Fig. 4a (see also Fig. S15, ESI†)—as a consequence of their consumption *via* a hydrogenation reaction during treatment in flowing H_2_ at 313 K and 1 bar.^55,56^ The bands described above represent species formed from ethylene on the cluster by self-hydrogenation (*vide infra*).^57,58^ During treatment of the sample in flowing H_2_, these subtraction bands increased, as a result of continuing consumption of bound ethyl ligands *via* hydrogenation. Concomitantly, ethane was detected in the effluent gas by mass spectrometry (Fig. S16†). Fig. 4b demonstrates the dynamics of the loss in intensity of the IR band at 2959 cm^–1^, which implies that the ethyl ligands represent the stable intermediate present during ethylene hydrogenation. The kinetics represented by data in Fig. 4b are governed by the characteristic time scale for the half hydrogenation of bound ethyl ligands-multiple turnovers in this experiment were impossible because of the lack of an ethylene coreactant in the gas feed. To measure an apparent activation energy characterizing this catalytic half-hydrogenation process, kinetics data were acquired with the sample in flowing H_2_ at 303 K and 313 K, and at a pressure of 1 bar (Fig. S25 and S26†); details follow.

### Ethylene hydrogenation catalysis

**Fig. 5:**
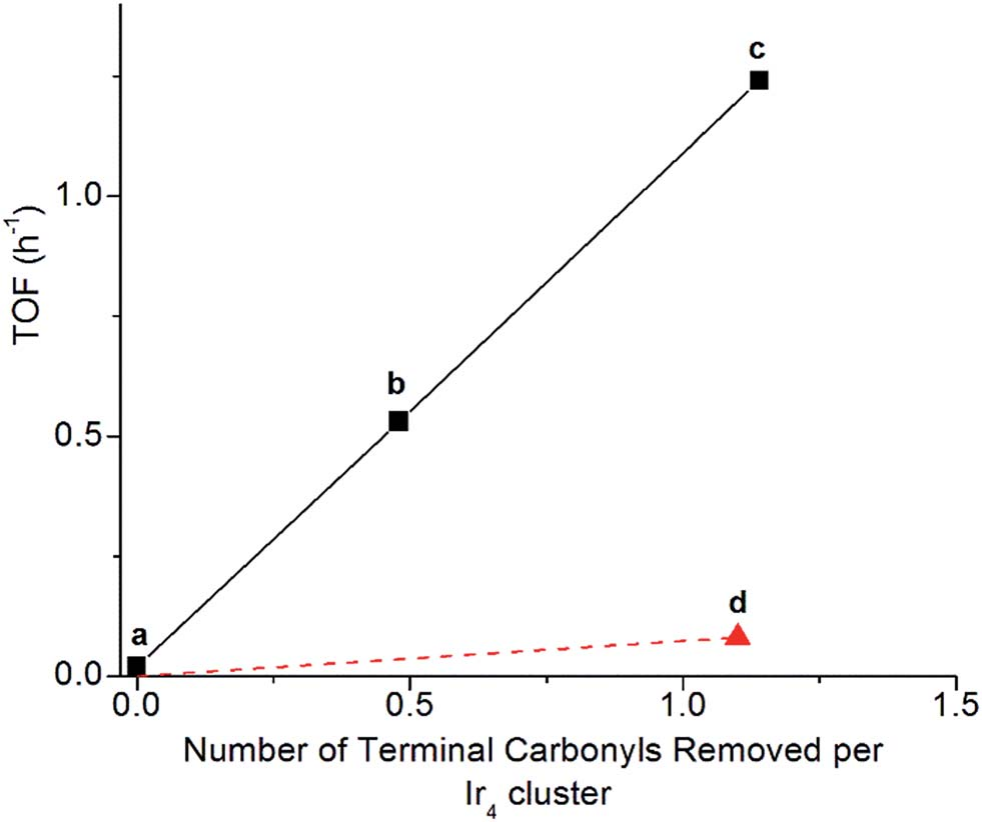
Dependence of catalytic activity represented as initial TOF for ethylene hydrogenation on the number of terminal carbonyl ligands removed per Ir_4_ cluster, for samples consisting of (a) cluster **1** (closed) supported on silica, (b) formed by TMAO treatment of cluster **1** in solution in absence of ethylene followed by supporting on silica, (c) formed by TMAO treatment of cluster **1** in solution with ethylene followed by supporting on silica, and (d) closed cluster **1** supported on silica treated in flowing H_2_. Quantification of carbonyl ligands was determined by the changes in band areas by IR spectroscopy with the sample in the solid state for (d) and in *n*-decane solution for (b) and (c). Catalytic hydrogenation reactions were conducted at 1 bar and 313 K with flow rates of 50 mL min^–1^ of helium, 10 mL min of H_2_, and 3.0 mL min^–1^ of C_2_H_4_; the catalyst mass was 250 mg, and the Ir content was 1.0% based on mass.

Clusters of **1** treated with TMAO, either with or without ethylene present, and subsequently supported on partially dehydroxylated porous silica provided an opportunity to investigate how the locations of sites where CO removal occurred in **1** influenced activity of the supported catalyst. Thus, we compared the catalysts using ethylene hydrogenation in a flow reactor as a test reaction. Fig. 5 shows the steady-state activities (extrapolated to initial time on stream to account for slight transients in Fig. S17†) per total Ir atom at 313 K. The data show that the silica-supported catalyst consisting of the cluster synthesized in the presence of an ethylene atmosphere is twice as active (point (c) in Fig. 5; turnover frequency, TOF = 1.2 h^–1^) as the one synthesized without ethylene (point (b) in Fig. 5; TOF = 0.53 h^–1^). For the former catalyst, reaction orders of 0.66 in H_2_ and –0.27 in ethylene were measured at the same total pressure and temperature, by varying reactant partial pressures separately in flowing helium (Fig. S23†). The apparent activation energy was also measured for the supported catalyst represented by point (c) in Fig. 5, corresponding to ethylene hydrogenation under steady-state conditions; the value is 64 kJ mol^–1^ (Table S3 and Fig. S27†).

Another sample was synthesized by removing CO ligands after supporting the cluster on silica, by treating supported **1** with H_2_ (flowing at 10 mL min^–1^, 313 K, and 1 bar for 20 h; IR spectra and catalyst performance data are shown in Fig. S18, S19 and Table S2†). During treatment of supported **1** with H_2_, IR spectra were measured, and they demonstrate the growing in of a band at 2110 cm^–1^ (Fig. S20†), which is assigned to a bound hydride on Ir and which reversibly disappears and reappears upon pulsing of D_2_ and H_2_, respectively (Fig. S21†). The decreasing IR intensity of terminal and bridging CO bands along with their blue shift suggests replacement of those CO ligands of supported **1** with hydride *via* oxidative addition of hydrogen (Fig. S21 and S22b†). In contrast to the aforementioned supported Ir_4_ cluster catalyst synthesized from **1** by oxidative decarbonylation *via* TMAO treatment, this one was found to have almost negligible activity for ethylene hydrogenation (point (d) in Fig. 5), even though 1.1 terminal CO ligands and 1.9 bridging CO ligands per cluster had been removed (Fig. S18†). Rapid rebonding of CO to all of these sites was observed, demonstrating the lack of any irreversible changes to the cluster during these H_2_ treatments, up to a temperature of 343 K (Fig. S22†).

## Discussion

### Uniqueness of isolated catalytic sites in supported tetrairidium cluster

Central results of this work are those demonstrating that (a) the Ir_4_ cluster frame with three bulky calixarene phosphine ligands bonded to basal-plane Ir atoms incorporated CO ligands that are not fluxional; (b) CO ligands can be removed selectively from Ir sites on the cluster located principally at terminal sites and, in an alternative treatment, bridging CO ligands can be removed selectively from the basal plane; and (c) the sites that incorporate ethyl ligands as a result of the synthesis are catalytically active for ethylene hydrogenation, whereas the basal-plane sites are not.

These results raise several fundamental questions that go beyond any addressed before, which we address in this section:

(1) How does the synthetic chemistry allow selective formation of distinct sites on the Ir_4_ cluster?

(2) What reactive intermediates form on the cluster, and how do they compare with those formed on extended metal surfaces under similar conditions?

(3) How does the mechanism of ethylene hydrogenation catalysis on the cluster compare with mechanisms of that reaction on metal surfaces?

(4) How can one explain the degree of uniformity of the catalytically active sites on the clusters, realizing the contrast between those sites and the intrinsically nonuniform sites that are present in almost all of the reported single-site (site-isolated) supported metal catalysts?

(5) How do the observations of reactivity of the catalytically active sites and catalytically inactive sites on the cluster compare with the poorly understood sites on metal surfaces that are represented with the designations “*” and “S”?

(6) How are the uniquely active sites on the clusters formed and how can we understand the mechanism of their formation?

These questions provide a rough outline of the Discussion section that follows.

### Chemistry of synthesis of sites with ethyl ligands on tetrairidium clusters and comparison with chemistry on metal surfaces

The data shown in Fig. 3 and Table 1 demonstrate the crucial role of ethylene present during TMAO treatment in determining where cluster decarbonylation occurs, leading to the formation of ethyl ligands that persist upon anchoring of the clusters to silica (Fig. S15 and Table S1†). The data show that the presence of ethylene during TMAO treatment leads to cluster decarbonylation predominantly at sites where terminal CO ligands are bound, which include apical sites. This decarbonylation chemistry is contrasted with that observed with TMAO in the absence of ethylene, because then decarbonylation occurs predominantly at sites with bridging CO ligands, all of which are located in the basal plane. Because formation of bound ethyl ligand takes place on metal surfaces from ethylene—by its self-hydrogenation (*i.e.*, hydrogenation of ethylene with hydrogen provided by ethylene^56–60^)—we suggest that self-hydrogenation similarly occurs in the reaction of **1** with TMAO in the presence of ethylene, to synthesize cluster-bound ethyl ligand, and that this reaction can occur only on specific sites of the cluster where bonding of ethyl as a ligand is favoured.

### Further comparison of tetrairidium clusters and metal surfaces: reaction intermediates

Besides bonding of ethyl ligand to the cluster, the data shown in Fig. 4a include no evidence of dehydrogenated surface species, such as ethylidyne, that one might have expected to form from ethylene following TMAO treatment in the presence of an ethylene atmosphere. Nor is there evidence of σ-bonded or π-bonded ethylene ligands on the supported cluster, which would have been evidenced by bands at frequencies above or near 2970 cm^–1^.^56,57,59,60^ The absence of such species on the cluster is in line with a previous observation, made after sequential reactive decarbonylation of **1** and its anchoring onto silica, followed by a treatment in ethylene.^44^ The lack of evidence of ethylidyne (or other bound dehydrogenated hydrocarbon species) is contrasted with reports of these species on extended noble metal surfaces—for example, ethylidyne forms even in the presence of H_2_, under conditions of steady-state ethylene hydrogenation catalysis.^31,57,59,61–63^ This difference in reactivities of the cluster and metal surfaces may be an indication that, because of their small size and extensive ligation,^39^ our Ir_4_ clusters lack the metal sites needed for accommodating bound dehydrogenated species, such as those that are readily observed on metal surfaces.

### Kinetics of ethylene hydrogenation and implications regarding reaction mechanism

The dynamic IR spectra shown in Fig. 4 represent a single hydrogenation reaction event on a bound ethyl fragment, corresponding to the second half hydrogenation of surface-bound ethyl to give gas-phase ethane. These data lead to an estimate of the average time for a single hydrogenation of Ir-bound ethyl to ethane of 15 ± 3 min at 313 K (see ESI† for details of the calculation). Under steady-state ethylene hydrogenation catalysis conditions, the cluster site represented by point (c) in Fig. 5 requires an average time of 13.2 min to turn over at 313 K, as shown in Fig. 5. The kinetics data corresponding to the experiments in Fig. 4 and 5 indicate a reaction order near zero in ethylene, corresponding to a quasi-equilibrated bound ethyl intermediate—and the data correspond to the same partial pressure of H_2_, making the TOF comparison between transient and steady-state experiments rigorously valid. The measured apparent activation energies for the single-hydrogenation reaction and the steady-state ethylene hydrogenation reaction are both 15 kcal mol^–1^. These data (Fig. S27†) are broadly in agreement with data representing other ethylene hydrogenation catalysts.^27,28,30,59^

These essentially equal characteristic times of reaction and apparent activation energies characterizing the transient single-hydrogenation reaction and the steady-state catalysis support the inference that the second half-hydrogenation of cluster-bound ethyl to give ethane shown by data in Fig. 4 is the rate-limiting process in the steady-state catalytic hydrogenation represented by the data of Fig. 5. This conclusion is similar to conclusions drawn for examples of hydrogenation catalysis on noble metal surfaces.^59,64–67^ Previously, a similar comparison of characteristic times for transient single-reaction events and for reaction under steady-state conditions has been made to elucidate the reactive intermediates involved in CO oxidation on Au/TiO_2_ catalysts.^68^

### Evidence of uniform catalytic sites on tetrairidium clusters

A crucial point is that the equivalence of the kinetics for the transient single-hydrogenation reaction experiment in Fig. 4 and the steady-state experiment as represented by point (c) in Fig. 5 requires that each site that is accessed in the single-hydrogenation reaction experiment contributes equivalently to the catalytic reaction rate under steady-state conditions. This conclusion supports the above-stated inference of the uniformity of the sites of our supported cluster catalyst and permits us to rule out the possibility that a small fraction of sites is responsible for catalysis under steady-state conditions of Fig. 5, following TMAO treatment. The evidence that all of these sites are equivalent catalytically shows that they are almost unique among supported metal catalysts. We regard these sites on the Ir_4_ clusters as site-isolated, which can be explained by the almost identical groups bonded to them—the catalytic sites are essentially supported on the triangle of basal-plane Ir atoms, which are ligated by bulky calixarene phosphine ligands.

### Evidence of “*” and “S” sites: linking hydrogenation catalysis on tetrairidium clusters and on metal surfaces

The data presented here demonstrate a strong analogy between the Ir_4_ clusters and extended surfaces of metals as hydrogenation catalysts, providing a foundation for understanding of the “*” and “S” sites—now in terms of well-characterized sites on a molecular catalyst. Recall that the catalytically active sites on surfaces correspond to the “*” sites, and the “S” sites correspond to sites that are not fruitful in hydrogenation catalysis. Recognizing an analogy between the metal cluster catalyst and the noble metal surfaces, we see that the sites for both the single-hydrogenation reaction kinetics of Fig. 4 and the steady-state turnover kinetics of Fig. 5 are “*” sites and the basal-plane sites are “S” sites. Recall that upon attempting ethylene hydrogenation catalysis on the cluster treated to incorporate hydride ligands, almost no catalytic activity was observed, as shown in point (d) of Fig. 5. Our measurements indicate that H_2_ treatment of silica-supported **1** leads to decarbonylation but that it is not fruitful in the sense of not synthesizing “*” sites previously occupied by apical CO at 40 °C, instead removing basal-plane CO to synthesize “S” sites, to which hydrogen bonds non-competitively in the presence of ethylene and to which CO freely rebonds to synthesize silica-supported **1** again. We further infer that the lack of ethylene bonding sites precludes a silica-supported **1** after hydrogen treatment from being an active ethylene hydrogenation catalyst, notwithstanding substantial removal of bridging and terminal CO ligands.

In contrast to synthesis of “S” sites, synthesis of “*” sites occurs by treating **1** with TMAO and takes place by a mechanism whereby CO dissociation is not rate limiting. Instead, nucleophilic attack of TMAO has been inferred to take place by transfer of oxygen lone pairs onto the carbon of the departing CO ligand, in a concerted fashion with O transfer from TMAO, *via* an SN_2_ mechanism^69,70^ This mechanism explains why selectivity for CO removal can be completely different from that in reactions requiring CO dissociation as the rate-limiting step, such as thermally driven decarbonylation with a sweep-gas treatment of supported **1**.^48–51,57,69–71^

With oxidative decarbonylation either in the presence or absence of ethylene, some apical CO is removed to synthesize “*” sites, as shown by the bands characterizing bound ethyl in the IR spectra of Fig. 4a and the catalytic activity for points (b) and (c) in Fig. 5. Data allowing discrimination between decarbonylation with TMAO at one of the two possible sites for terminal CO ligands, corresponding to either basal-plane or apical locations, are presented in Fig. 5, which indicates the degree of removal of apical CO as a function of total terminal CO removal—with catalytic ethylene hydrogenation activity used as a proxy for the former. This calculation is enabled by our assignment of the apical site as a “*” site, which the IR spectra show bonds to ethylene—and bonding to ethylene was not observed for “S” sites synthesized *via* thermal decarbonylation involving simple CO dissociation, on the basal plane.^44^

The direct proportionality shown in Fig. 5 for points (a), (b), and (c) implies that the terminal CO removed by TMAO was selectively located in all of those instances at “*” sites—those at the apical position. The justification of this inference requires considering the contrary scenario and showing that it leads to a contradiction: were some basal-plane terminal “S”-site carbonyls removed by TMAO corresponding to points (b) and (c) of Fig. 5, then the curve connecting points (a), (b), and (c) in Fig. 5 would be concave-up, in contrast to what was observed, because the ratio of “*” to “S” terminal carbonyls removed would increase for point (c) relative to point (b) in Fig. 5, for the same reason why the fraction of basal-plane bridging carbonyls decreased for point (c) relative to point (b) in Fig. 5.

In summary, reactive decarbonylation with TMAO in the presence of ethylene produces 1.1 “*” sites per cluster located at the apical position and 0.33 “S” sites located in bridging positions of the basal plane. This result is in contrast to what was observed for reaction in the absence of ethylene, which led to 0.48 “*” sites and 0.81 “S” sites per cluster. The linearity of Fig. 5 confirms the catalytic equivalency of each of these “*” sites, bolstering the results demonstrated by the equivalence of the kinetics in the single-hydrogenation reaction and in steady-state catalysis.

### How ethylene dials in the synthesis of active “*” sites

We hypothesize that the mechanism by which ethylene leads to the synthesis of “*” rather than “S” sites during TMAO treatment relies on reversibility, whereby, in the presence of ethylene, the system finds the thermodynamic sink of bonding ethyl to an apical “*” site. Such a mechanism is facilitated by a thermodynamic driving force of ethylene bonding at the apical position to steer decarbonylation to occur there selectively when TMAO treatment is performed in the presence of ethylene. This mechanism leverages on our previous observation^44^ of apical sites as those on the cluster that are unique in their ability to bond ethylene stably, as supported by electronic structure calculations. Implicit to this mechanism occurring with TMAO treatment in the presence of ethylene is microscopic reversibility^72,73^ and quasi-equilibration of the TMAO reaction with CO ligands in the basal plane of **1**, which allows the steering to the lowest-energy configuration, consisting of bound ethyl at the apical position. Were decarbonylation with TMAO not microscopically reversible, the presence of ethylene would have no effect on the ratio of terminal to bridging CO vacancies, because there is no plausible mechanism involving a three-body encounter of TMAO, ethylene, and bound CO, meaning ethylene would simply be a spectator in the process of CO removal. Thus, we infer that there must be a degree of reversibility in the CO oxidation by TMAO—so that the system has a means of going back when, because of the kinetics, less-favored sites are opened. These less-favored sites that do not offer the energetic benefit of ethylene bonding are the “S” sites located on the basal plane.^44^

Therefore, to explain our reaction orders in ethylene hydrogenation catalysis (Fig. S23†), we invoke a two-site model of our supported cluster catalyst, consisting of apical “*” sites for bonding and activating ethylene, and basal-plane “S” sites where hydrogen and CO bond, but not in competition with ethylene, in a quasi-equilibrated fashion.^29–31^ Earlier workers, employing “*” and “S” sites to explain data observed with a supported metal catalyst (Pt/SiO_2_), invoked similar two-site models to account for reaction orders of 0.67 in H_2_ (at 273 K and 100 mbar of ethylene) and –0.17 in ethylene (at 298 K and 200 mbar of H_2_) at 273 K. Such kinetics cannot be obtained from the classic Horiuti–Polanyi mechanism of metal-catalyzed ethylene hydrogenation, which predicts a first-order dependence in H_2_ partial pressure under similar conditions (a H_2_ reaction order of unity indicates competitive adsorption of both ethylene and hydrogen on the same site).^29–31^ The previously mentioned near one-half reaction order for H_2_ and zero for ethylene are close to the values characterizing our supported cluster catalyst that were measured under similar conditions. Thus, we infer that our substituted Ir_4_ cluster is a representative model of more typical supported noble metal catalysts for hydrogenation and related hydrogen-transfer reactions, which invoke “S” sites as crucial non-competitive (with respect to hydrocarbon ethylene) sites for bonding hydrogen to explain low-temperature hydrogenation kinetics. For completeness, we note that not all supported clusters consist of “S” sites. Previously reported triosmium carbonyl clusters on silica exhibit kinetics that are consistent with the classical Horiuti–Polanyi mechanism, in which H_2_ and ethylene compete for all metal sites (*i.e.*, the catalytic reaction is nearly zero order in ethylene and first order in hydrogen).^9^ What makes the connection between reaction orders and “S” sites so compelling in the work reported here is that there is a clear and consistent molecular explanation for the observed kinetics involving selective molecular recognition by basal Ir atoms of the silica-supported cluster. The physical origin of this selectivity is electronic, not steric.^44^

## Conclusions

Knowing that removal of CO ligands from the Ir_4_ cluster can occur at the apical position with TMAO treatment, we have controlled the locations of open sites on the clusters resulting from CO oxidation by TMAO—and counted them. On the basis of the liquid-phase IR data of Fig. 3, combined with the result that the ratio of terminal to bridging CO ligands is not altered by anchoring of the clusters to silica, we draw the following conclusions: (i) approximately 37% of open sites synthesized by TMAO treatment in the absence of an ethylene atmosphere are located at the apical Ir atom—leading to a catalytically productive site for ethylene hydrogenation; (ii) in contrast, in the presence of an ethylene atmosphere, this synthesis leads to approximately 78% of open sites at the apical Ir atom; and (iii) both of these conclusions demonstrate a clear benefit over thermally driven decarbonylation, which leads to virtually no open sites at the apical Ir atom. This selective synthesis of a single-site metal catalyst within an isolated and uniform environment has been achieved with a metal cluster—and metal polyhedra comprising clusters, intrinsically, are expected to exhibit heterogeneous distributions of reactive sites (Fig. 1). The synthesis reported here overcomes the expected heterogeneity because the synthesis chemistry allows dialing in of the productive catalytic sites—a remarkable degree of catalyst control. The sites that could bond to the reactant ethylene during their opening with TMAO (and do catalysis) are the ones synthesized in preponderance, in contrast to what was observed when sites were opened in the absence of ethylene. We posit that this approach to control of single-site reactivity may open a door to the more general control of the synthesis of catalytic sites on metal surfaces, by choice of ligand atmospheres—for dialing in the opening of sites with unique reactivity and selectivity.
