# Supplementary material for: Dialing in single-site reactivity of a supported calixarene-protected tetrairidium cluster catalyst
Source: Chem Sci. 2017 May 4;8(7):4951–60. doi: 10.1039/c7sc00686a (PMC5607854; doi:10.1039/c7sc00686a)
Supplement: Supplementary file 1 [file SC-008-C7SC00686A-s001.pdf]

Electronic Supplementary Material (ESI) for Chemical Science.
This journal is © The Royal Society of Chemistry 2017

Dialing in single-site reactivity of a supported calixarene-protected tetrairidium cluster catalyst

Andrew Palermo[a], Andrew Solovyov[b], Daniel Ertler[b], Alexander Okrut,*[b] Bruce C. Gates,*[a] Alexander Katz*[b]

[a]Department of Chemical Engineering, University of California at Davis, One Shields Avenue, Davis, California 95616, United States

[b]Department of Chemical and Biomolecular Engineering, University of California at Berkeley, Berkeley, California 94720-1462, United States

*Correspondance to: aokrut@berkeley.edu, bcgates@ucdavis.edu, and askatz@berkeley.edu

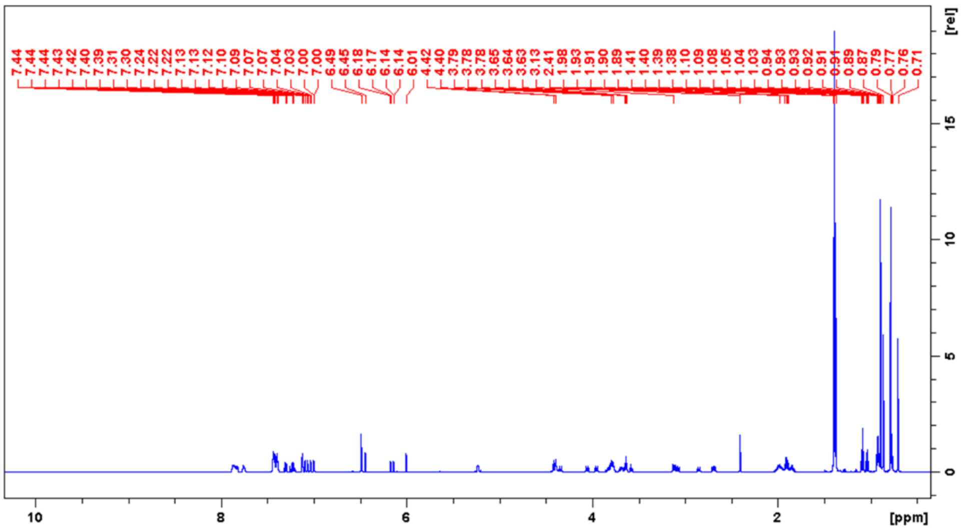

Figure S1. ^{1}H NMR spectrum (AV600, $CDCl_3$) of tetrairidium-tri-calixarene-phosphine cluster **1**

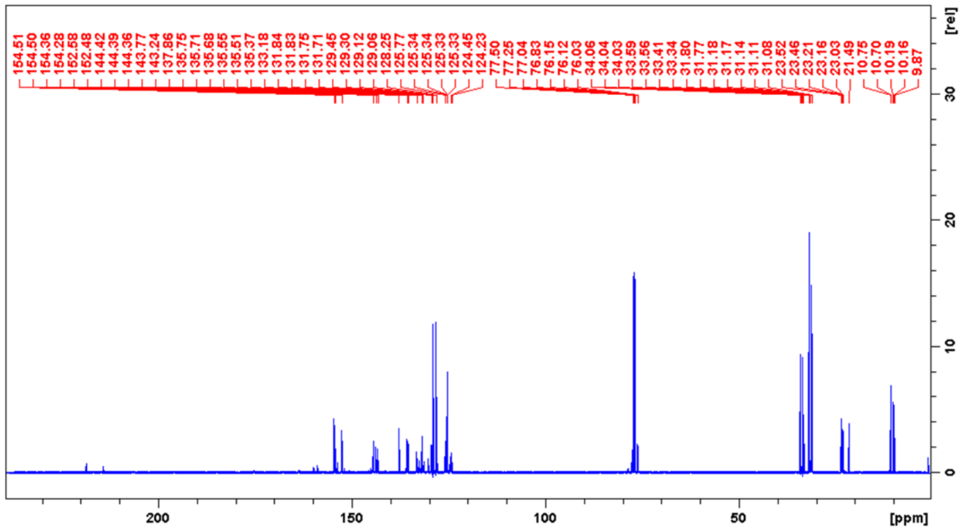

Figure S2. ^{13}C qNMR spectrum (AV600, $CDCl_3$) of tetrairidium-tri-calixarene-phosphine cluster **1**.

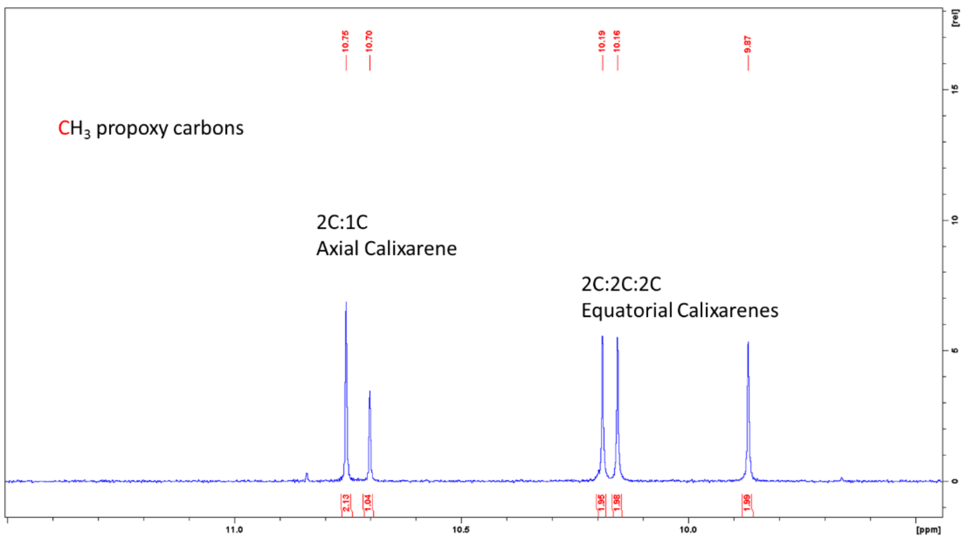

Figure S3. ^{13}C qNMR spectrum (AV600, $CDCl_3$) of tetrairidium-tri-calixarene-phosphine cluster **1** (CH_3 propoxy region).

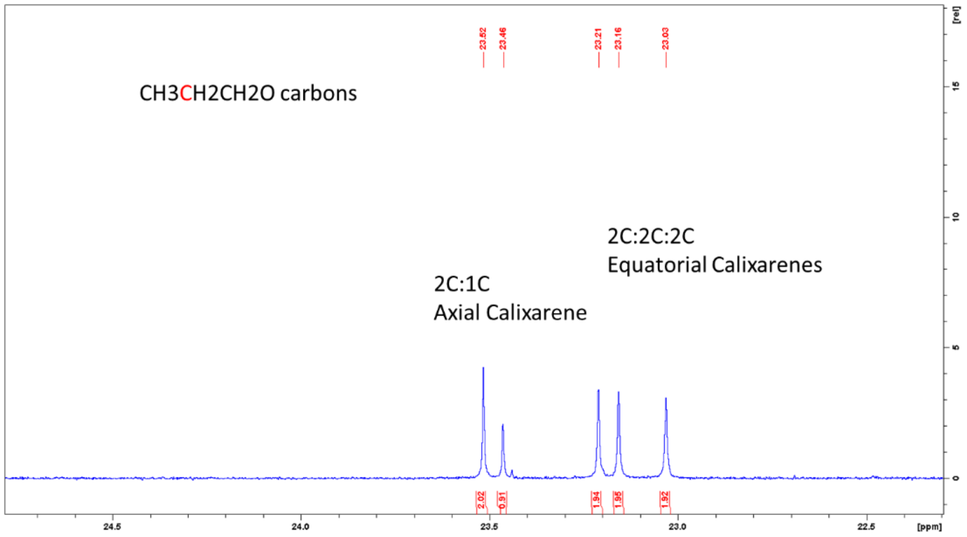

Figue S4. ^{13}C qNMR spectrum (AV600, $CDCl_3$) of tetrairidium-tri-calixarene-phosphine cluster **1** ($CH_3CH_2CH_2O$ region).

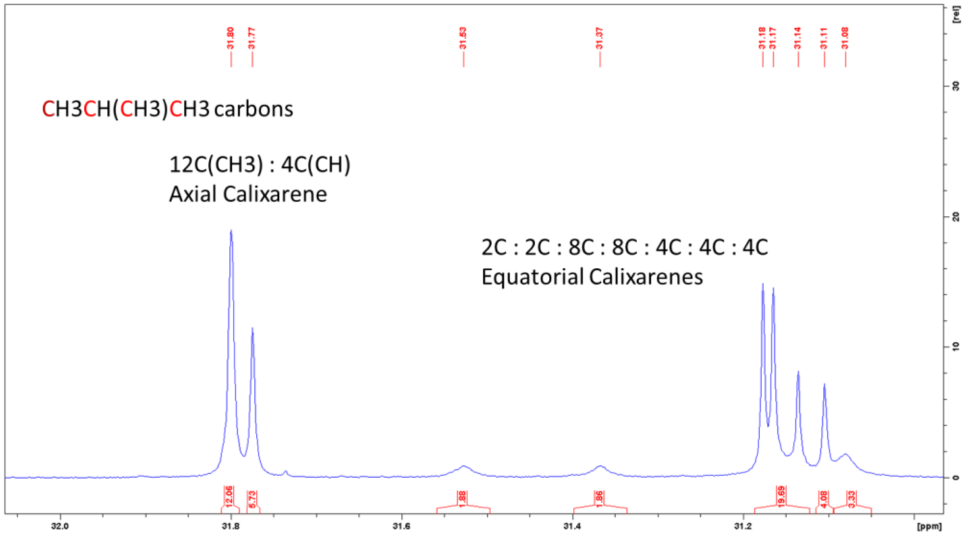

Figure S5. ^{13}C qNMR spectrum (AV600, $CDCl_3$) of tetrairidium-tri-calixarene-phosphine cluster **1** (*tert*-Bu region).

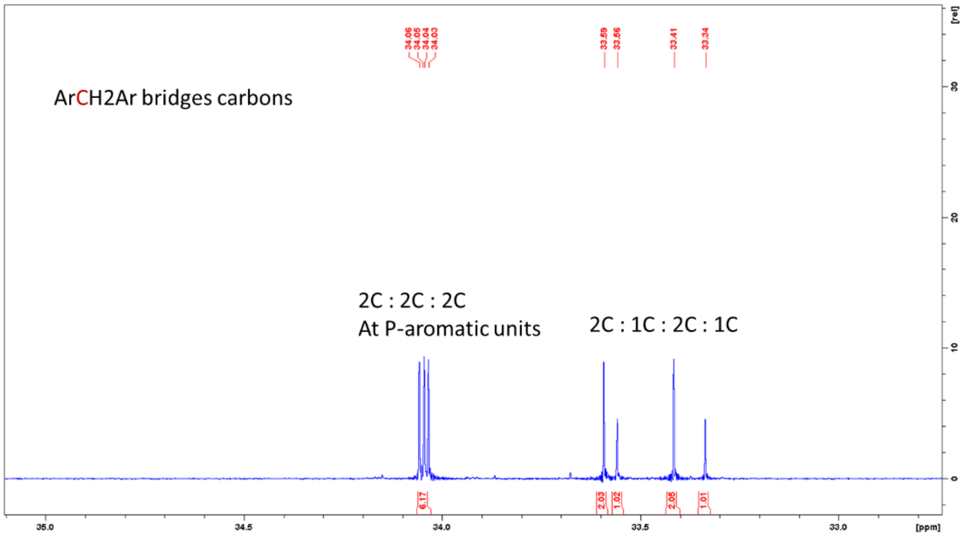

Figure S6. ^{13}C qNMR spectrum (AV600, $CDCl_3$) of tetrairidium-tri-calixarene-phosphine cluster **1** (CH_2 bridges region).

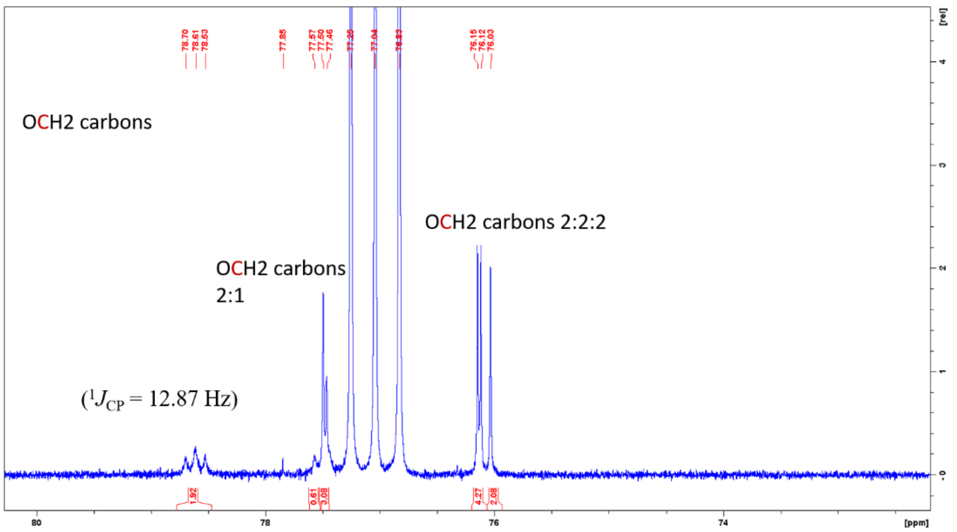

Figure S7. ^{13}C qNMR spectrum (AV600, $CDCl_3$) of tetrairidium-tri-calixarene-phosphine cluster **1** (*OCH_2* region).

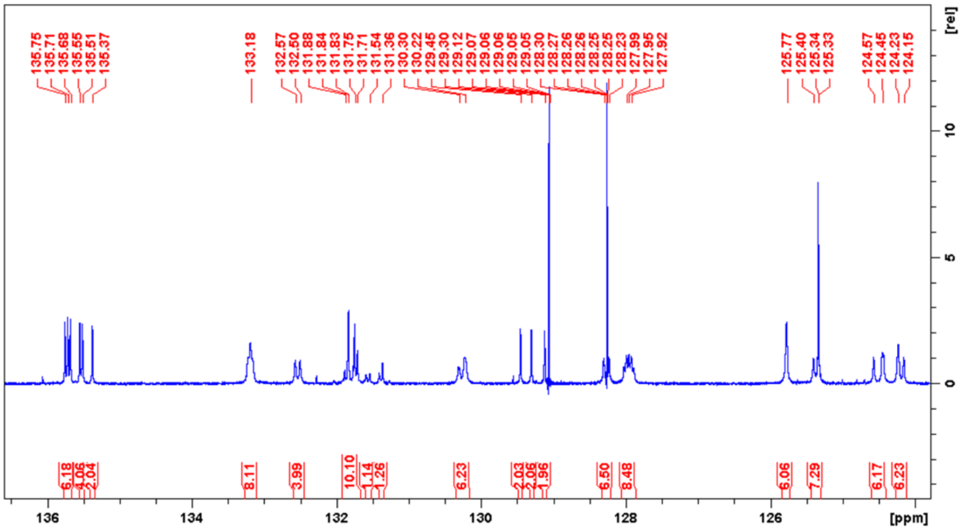

Figure S8. ^{13}C qNMR spectrum (AV600, $CDCl_3$) of tetrairidium-tri-calixarene-phosphine cluster **1** (*aromatic* region).

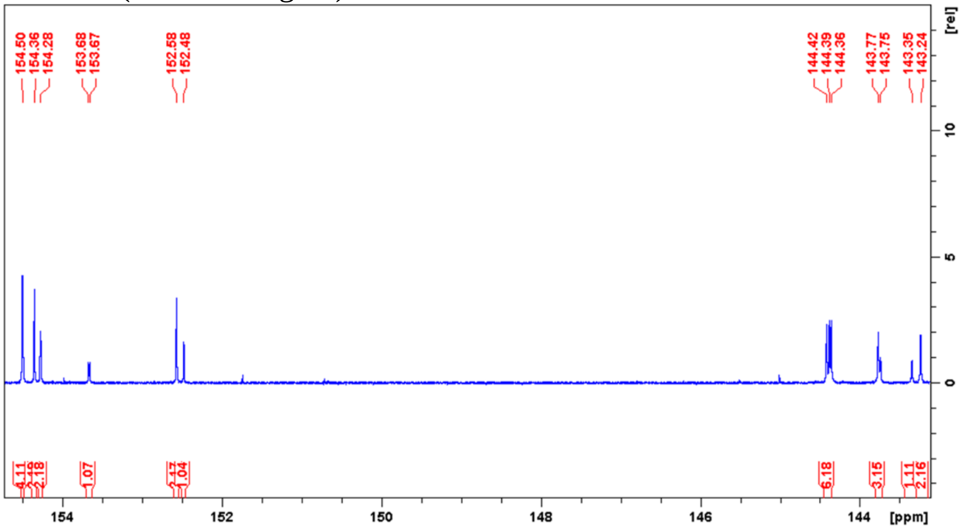

Figure S9. ^{13}C qNMR spectrum (AV600, $CDCl_3$) of tetrairidium-tri-calixarene-phosphine cluster **1** (*tertiary* aromatic carbon region).

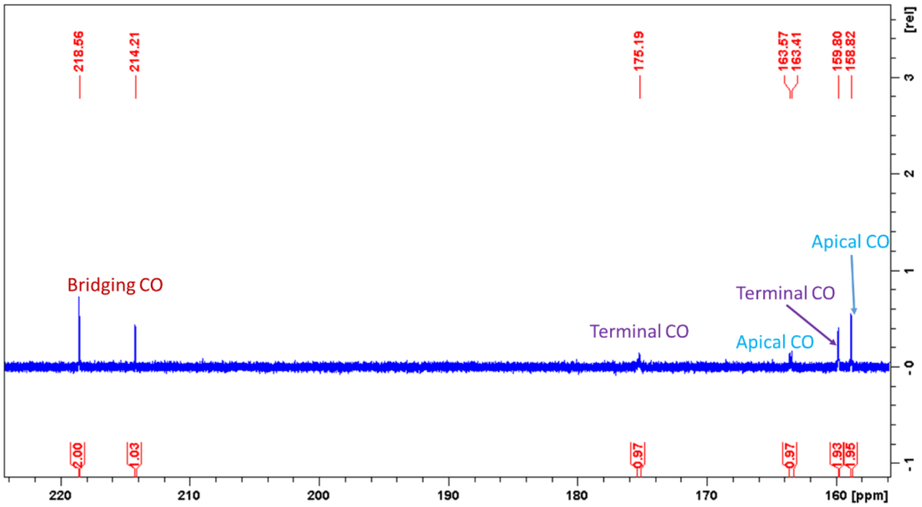

Figure S10. ^{13}C qNMR spectrum (AV600, $CDCl_3$) of tetrairidium-tri-calixarene-phosphine cluster **1** (carbonyl region). Terminal carbonyl at 175.19 ppm is equatorial. Terminal carbonyls at 159.80 ppm are axial.

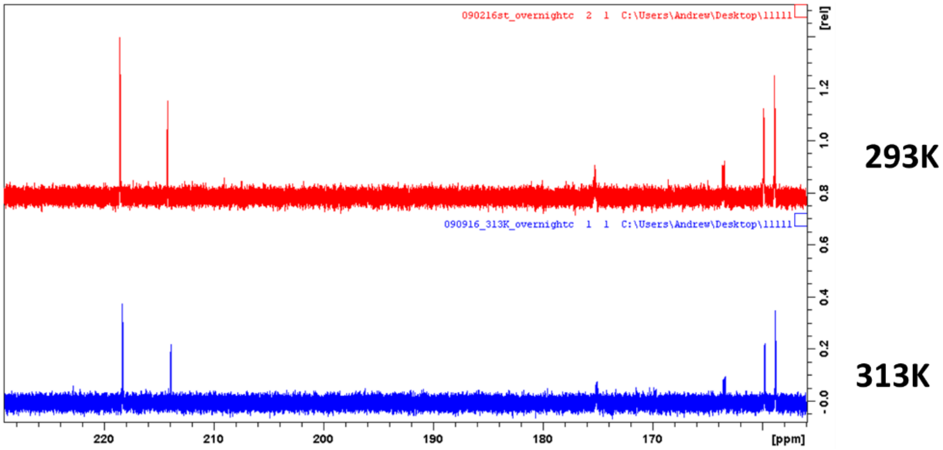

Figure S11. ^{13}C VT NMR spectra (AV600, CDCl3) of tetrairidium-tri-calixarene-phosphine cluster **1** (carbonyl region).

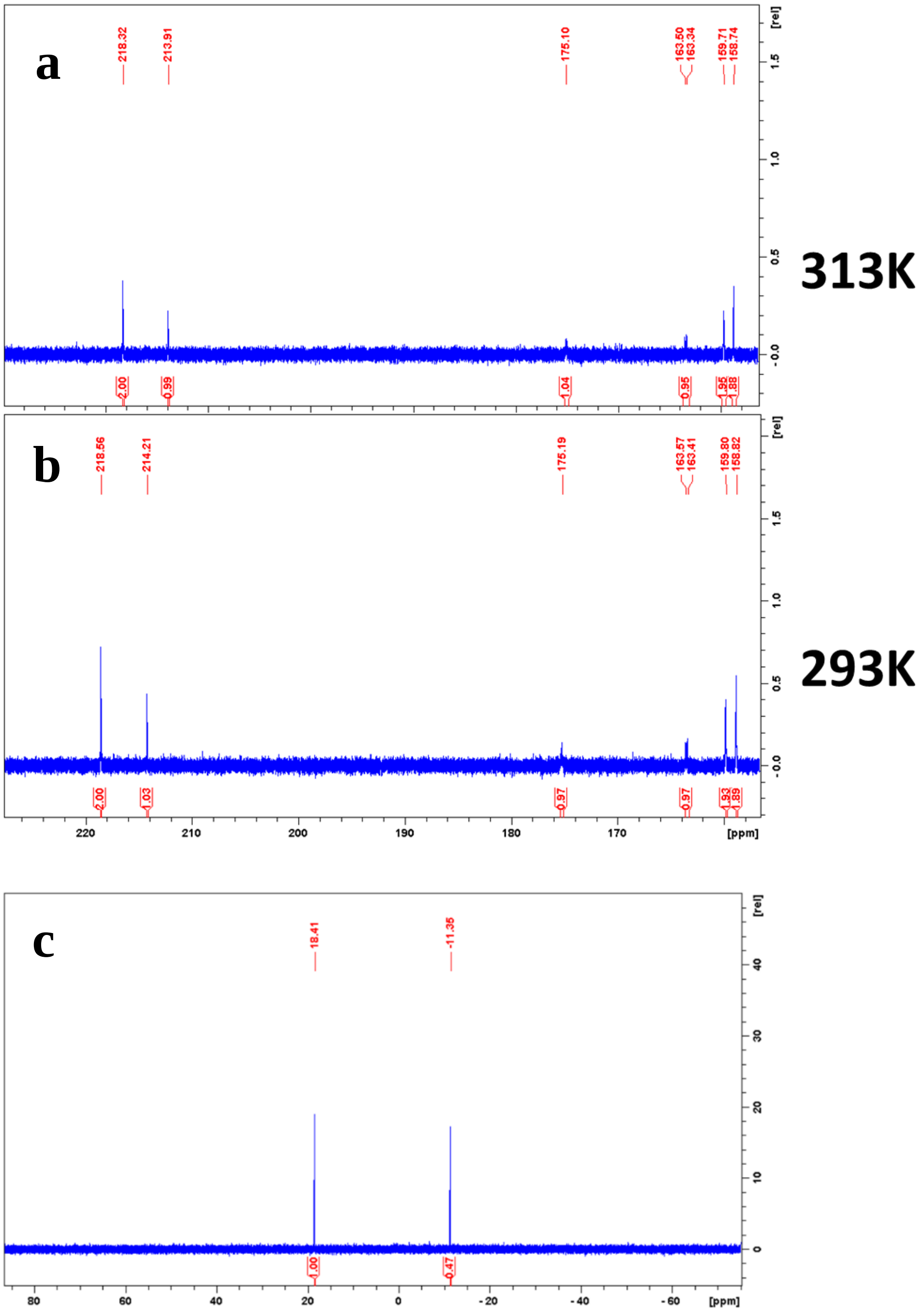

Figure S12. ^{13}C VT qNMR spactra (AV600, CDCl3) with integration values (carbonyl region) at (a) 313 K and (b) 293 K, and ^{31}P NMR spectrum (AV600, CDCl3) (**c**) of tetrairidium-tri-calixarene-phosphine cluster **1**.

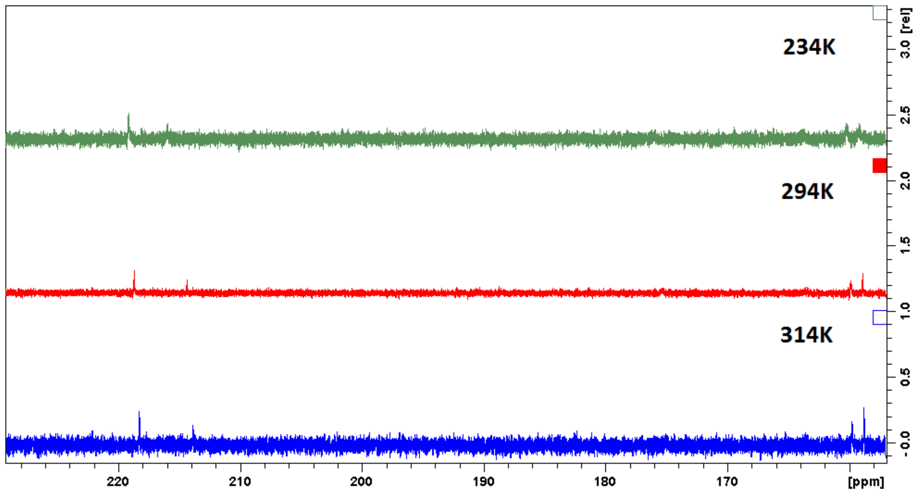

Figure S13. ^{13}C VT qNMR spactra (DRX500, CDCl3) of tetrairidium-tri-calixarene-phosphine cluster **1** (carbonyl region). All resonances in the ^{13}C NMR spectrum of cluster **1** become broader upon decreasing temperature to -40 ^{o}C, as a result of the decreasing three-dimensional conformational mobility (flattened cone-flattened cone interconversion of tetraalkylated calixarenes) of the calixarene skeleton.

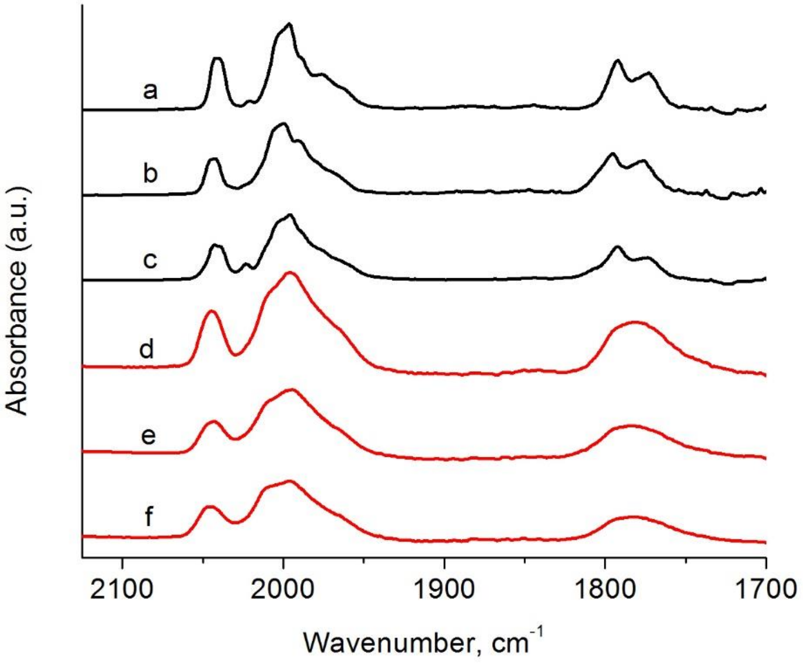

Figure S14. IR spectra comparing Ir_4 clusters in hexane (black) for (a) **1**, (b) **1** opened cluster by TMAO in an atmosphere of ethylene, and (c) **1** opened cluster by TMAO in an atmosphere of argon and supported Ir_4 clusters on SiO_2 (red) for (d) **1**, (e) **1** opened cluster by TMAO in an atmosphere of ethylene, and (f) **1** opened cluster by TMAO in an atmosphere of argon.

Table S1. Area ratios of terminal bands to bridging bands for Ir_4 characterizing selectivity of opening method.

Terminal/Bridge (Area)	Solution	Solid-State
Closed	2.42	2.42
TMAO C_2H_4	2.28	2.31
TMAO Ar	2.90	2.93

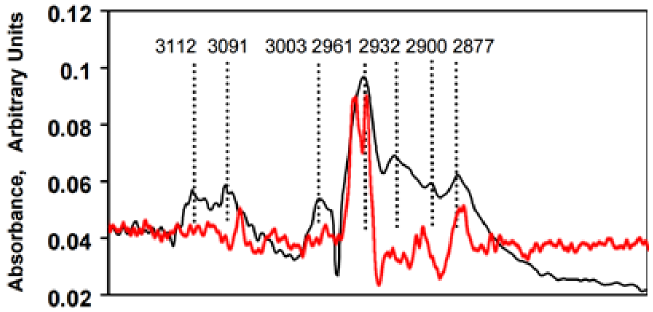

Figure S15. (Red) IR difference spectrum in the ν_{CH} region of cluster **1** opened with TMAO in an atmospher of ethylene, treated with H_2 (1 bar, 313 k, 10 mL/min) flow for 120 min, and the untreated TMAO (ethylene atmosphere) opened cluster **1** plotted over the work of Gates charactrizing ethyl species on open tetrairidium clusters.[S1] The lack of ethylidyne-based bands in this spectrum indicate no ethylidyne present in the system. Were this not the case, we would anticipate observing ethylidyne bands after approximately 30 min of H_2 flow, based on ethyl bands appearing at 10 min in Figure 1, and allowing for a factor of 3-fold slower kinetics for ethylidyne-based bands relative to ethyl, as reported previously by Frei et al.[S2]

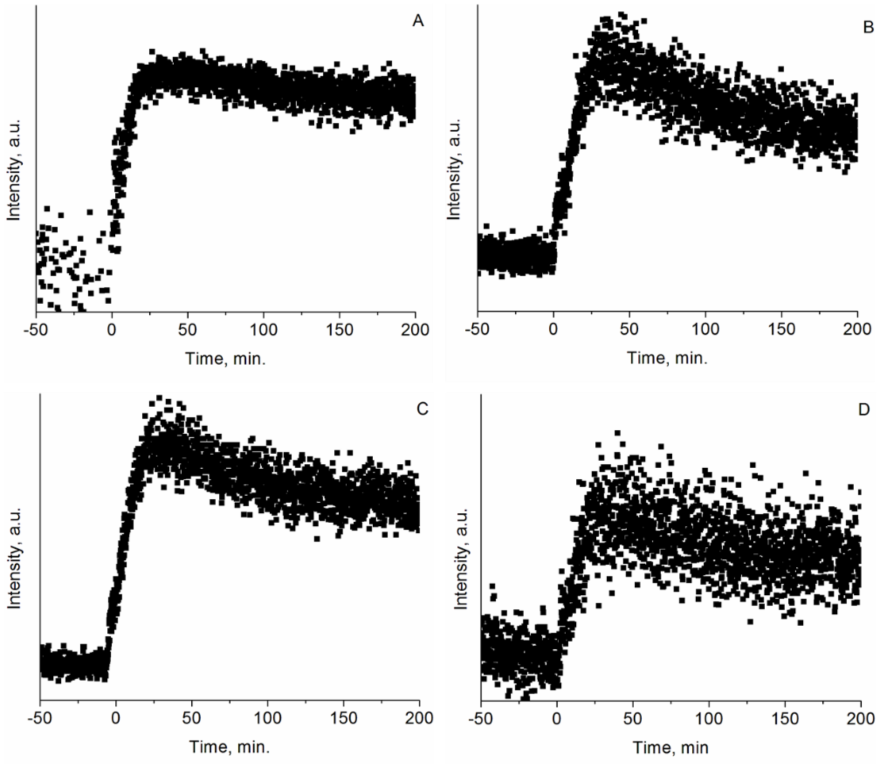

Figure S16. Time scales of mass spectrometry data for fragments (A) 26, (B) 27, (C) 29, and (D) 30 characterizing ethane production during H_2 treatment (313 K, 1 bar, 10 mL/min) of closed cluster **1** opened with TMAO in an atmosphere of ethylene.

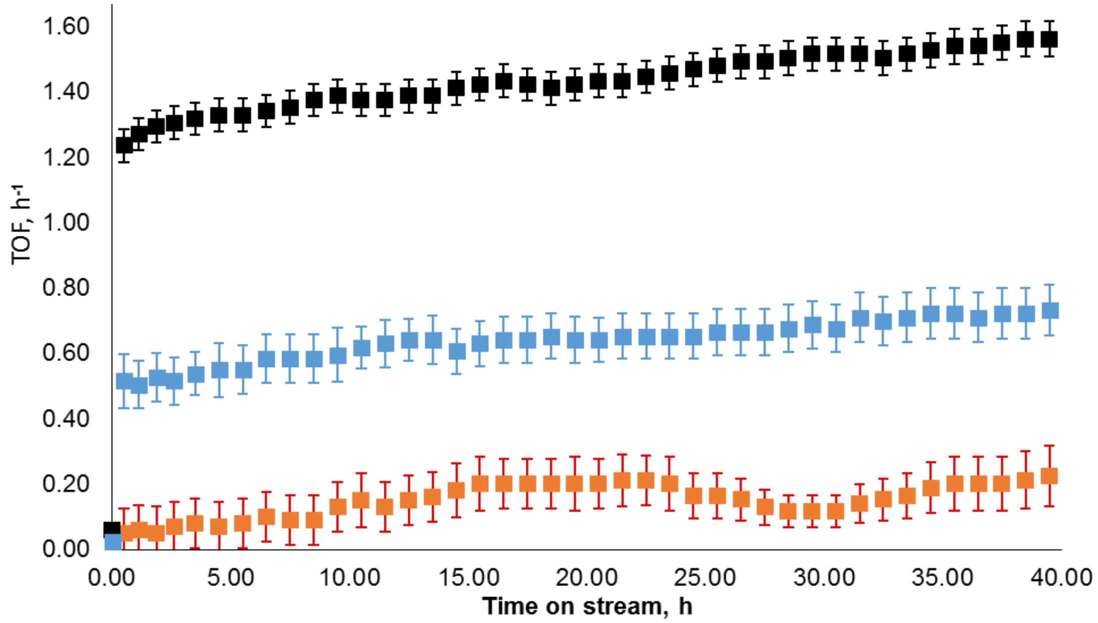

Figure S17. Ethane formed during ethylene hydrogenation at 313 K, atmospheric pressure, for closed cluster **1** (orange), TMAO open cluster **1** in Ar (blue), and TMAO open cluster **1** in ethylene (black) as a function of time on stream. Total flow rate was 63 mL/min (10 mL/min H_2, 3 mL/min C_2H_4, and a balance of helium).

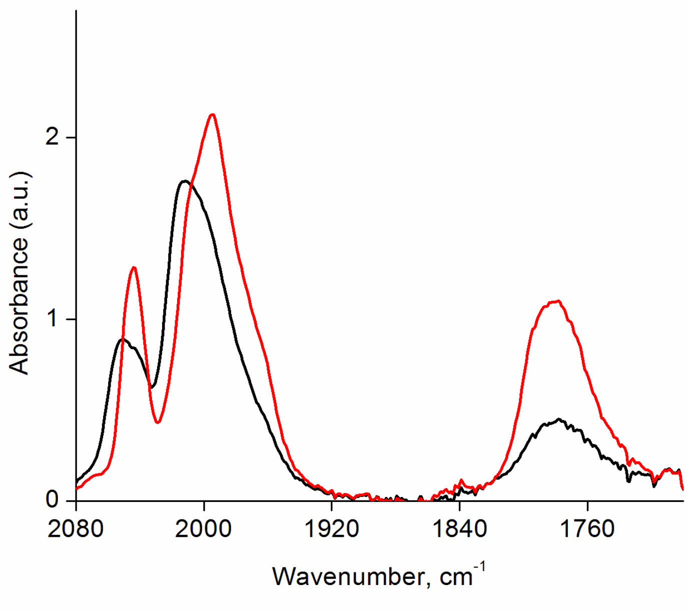

Figure S18. IR difference spectra of characterizing supported closed cluster **1** (red) that underwent 20 hours of H_2 / He treatment (10 mL/min of H_2, 53 mL/min of He, 313 K, 1 bar) (black). Difference spectra are a subtraction between the supported cluster and silica support.

Table S2. Realative change in area for terminal and bridging carbonyls when treating closed cluster **1** with flowing H_2 / He (10 mL/min of H_2, 53 mL/min of H3, 313 K, 1 bar).

Sample	Relative Terminal Carbonyl Area	Relative Bridging Carbonyl Area
Closed	1.0	1.0
H_2	0.82	0.37

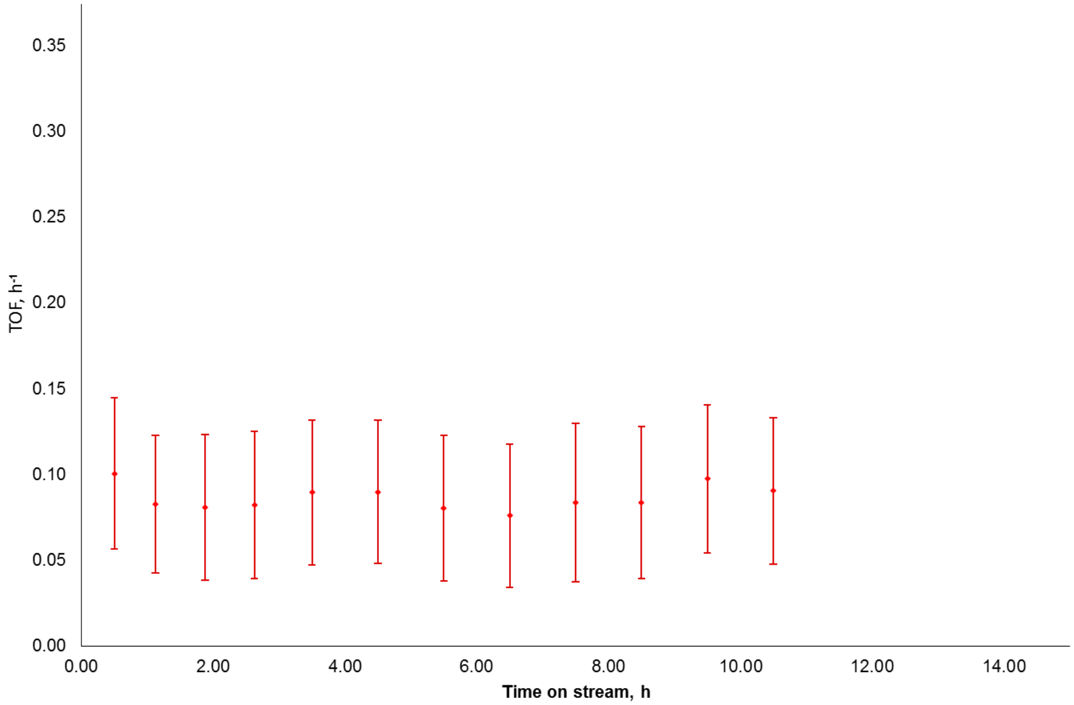

Figure S19. Ethane formed during ethylene hydrogenation, for closed cluster **1** supported on silica after the supported cluster underwent 20 h of flowing H_2 at 10 mL/min, 313 K, 1 bar. After H_2 treatment cluster underwent ethylene hydrogenation with a total flow rate was 63 mL/min, 313 K, 1 bar (10 mL/min H_2, 3 mL/min C_2H_4, and a balance of helium).

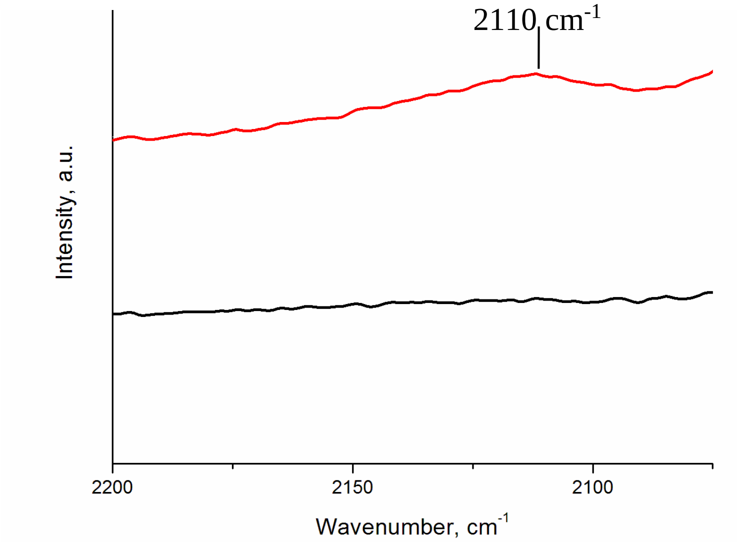

Figure S20. Difference spectra showing the growth of band at 2110 cm^{-1} characterizing Ir-H after 60 min (red) of H_2 flow at 313 K, 1 bar, 20 mL/min. This band is at the same position as a previously reported band for Ir-H stretch in a mononuclear complex,[S3] as well as the metal-H stretch for a Pt catalyst.[S4] Each spectrum is the difference between the hydrogen-treated sample and the sample prior to treatment. The black spectrum is a control, which represents the difference spectrum obtained before starting H_2 treatment and is the same as observed with D_2 treatment.

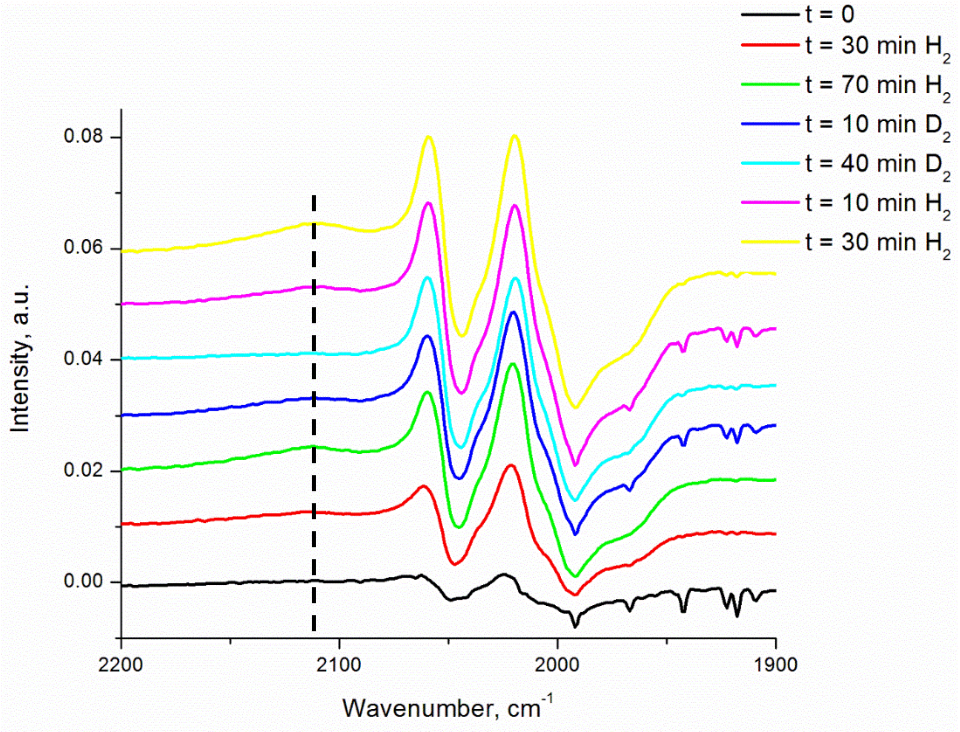

Figure S21. Difference IR spectra between showing the formation and removal of hydride bands. Each spectra is the difference between the catalyst that has underwent H_2/D_2 treated and supported cluster **1**. A vertical dashed line is drawn at 2110 cm-1 as an aid to the eye to visualize the band attributable to Ir-H. The observed changes in the carbonyl-band region demonstrates a blue shift as a result of oxidative addition and CO loss during H_2/D_2 treatment.

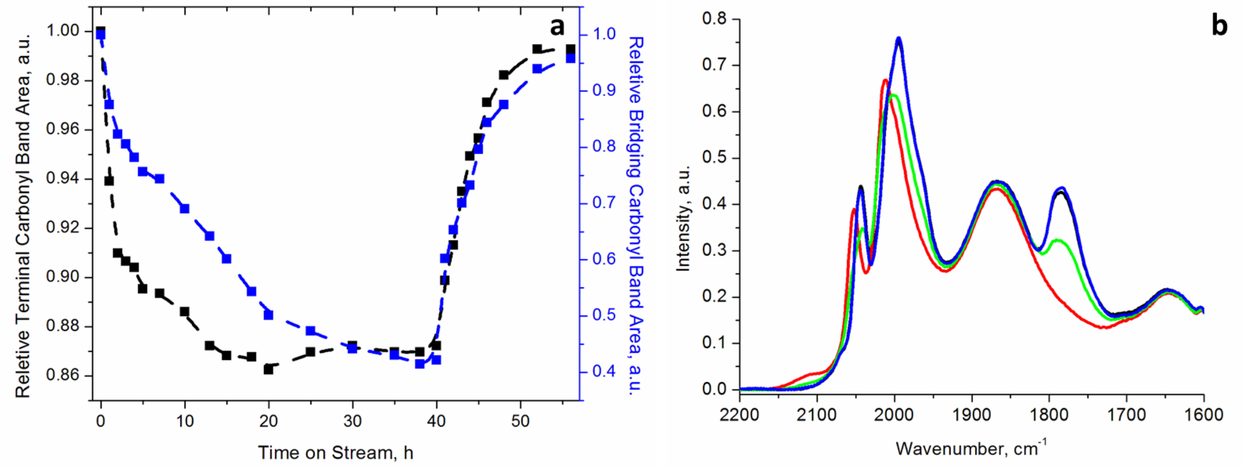

Figure S22. (a) Carbonyl-band area for terminal (black) and bridging (blue) CO ligands for **1** supported on dehydroxylated porous silica during 40 h of hydrogen treatment (10 mL/min) followed by CO treatment (20 mL/min) at 313 K and 760 torr. (b) Spectra characterizing the reversability CO loss and rebonding in silica-supported **1** during H_2 flow (10 mL/min, 760 torr) at variable temperatures followed by CO recarbonylation (20 mL/min, 760 torr) in a sequential treatment consisting of: (i) silica-supported cluster **1** at 293 K before hydrogen treatment (blue); (ii) decarbonylation at 313 K during H_2 treatment (green); (iii) decarbonylation at 343 K during H_2 treatment (red); (iii) recarbonylation by CO flow at 343 K (black). The green and red spectra represent the steady state decarbonylation at 313 K and 343 K, respectively, whereas the blue and the black are nearly overlayed on each other. The observed blue shift in the green and especially red spectra in the terminal CO region are a result of oxidative addition during H_2 treatment, as a result of replacing CO with hydride ligands.

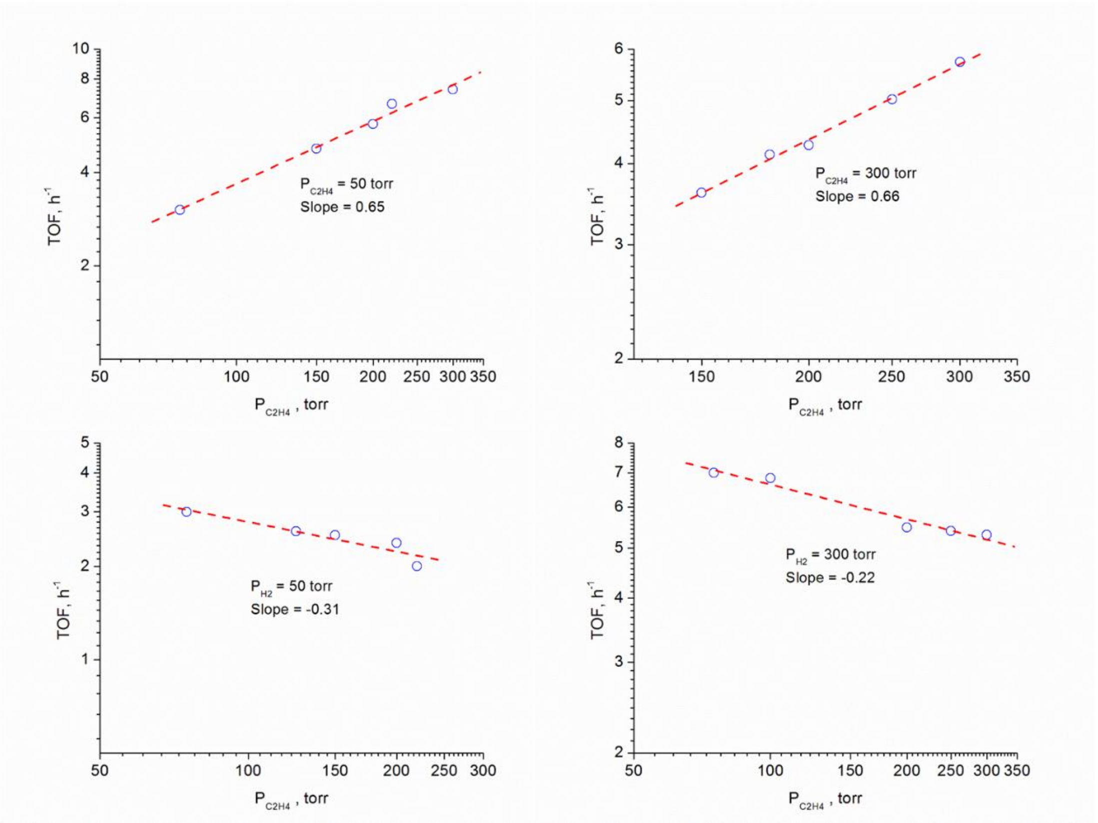

Figure S23. Effects of varying reactan partial pressures on ethylene hydrogenation catalystic activity at 343 K and 760 torr. Activity was calculated using a 1 wt% Ir sample of closed cluster 1, decarbonylated with TMAO in the presence of ethylene. Rate orders determined by varying the partial pressure one reactant while keeping the other constant (balance He).

Table S3. Variable temperature steady-state rate data and apparent activation energy for silica-supported catalyst consisting of closed cluster 1 decarbonylated with TMAO in an atmosphere of C2H4.

Material	Temperature, K	Ethane formation, ppm	Apparent activation energy, kJ/mol
$Ir_4L_3(CO)_9$	301	53	
decarbonlyated with	307	79	
TMAO in C_2H_4	313	136	64
	316	174	
	321	254	

Time Scale Derivation

The reported activity for cluster **2,** 313 K and 1 bar, (cluster **1** that has been decarbonylated with TMAO in an atmosphere of ethylene) is 1.14 h^{-1} per Ir atom. When looking at only the active cluster, one turnover will take 13.2 min as shown below.

$$TOF = (1.14)h^{-1} * Ir_{atom}^{-1}$$

$$= \frac{1.14}{h\ Ir_{atom}} * \frac{h}{60\ min} * \frac{4\ Ir_{atom}}{Ir_{4cluster}} = 0.0760\ (\mathrm{min})^{-1}$$

$$\frac{1}{TOF} = 13.2 \frac{min}{turnover_{cluster}}$$

In the following analysis we us the IR spectroscopy to find a near identical time scale for a single turnover experiment where we use the cluster described above and hydrogenate its ethyl ligand. Here we use the data in Figure A1, which has taken the data in Figure 2b and normalized to represent conversion, to identify an average timescale, τ, for the process. In Figure S23, there are two regimes a transient period and a steady state, shown below respectively.

$$y_{Transient} = 0.025x + 0.1534$$

$$y_{steadystate} = 0.0011x + 0.8623$$

$$x = 29.7\ min$$

The intersection of these two regimes will mark the end of the reaction at time t_f which is 29.7 min with an error of ± 6.31 min determine by the method of least squares.

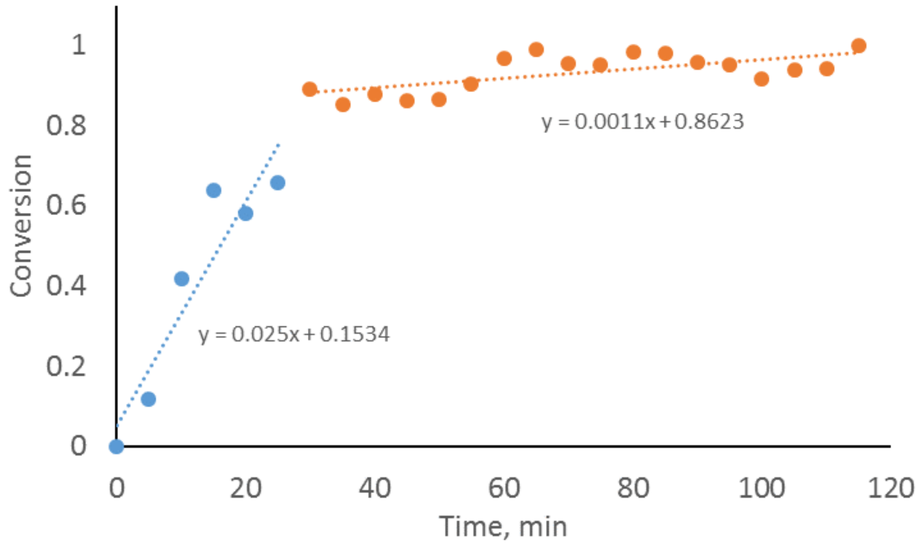

Figure S24. FTIR data of the removal of ethyl ligand at the ν_{CH} region, where intensity is plotted as conversion assuming all of the ethyl ligand has been removed at steady state. Flow conditions were: 1 bar, 313 K, He = 10 mL/min, H_2 = 10 mL/min.

In the analysis we view the conversion of ethyl ligand to ethane by function x(t), where N is the rate of ethyl conversion to ethane and N_{tot} is the total number of ethyl ligands bonded to the apical Ir atom.

$$x(t) = \frac{N}{N_{tot}}$$

From Figure S22, we model the conversion of ethyl ligand as a zero order rate law

$$N(t) = kt$$

At steady state where t = t_f

$$N_{tot} = kt_f$$

We define the average timescale, τ, as the average time the catalyst takes to complete a single turnover from t = 0 to the steady state time, t = t_f (where no or a negligible amount of ethyl ligands remain). This timescale is found by the rate of production of all events from t=0 and t=t_f. Shown by the average function value below.

$$f_{avg} = \frac{1}{b-a}\int_a^b f(x)dx$$

Substituting the appropriate values where f(x) is represented as conversion x(t)

$$\tau_{avg} = \frac{1}{t_f - 0}\int_0^{t_f} x(t)dt$$

$$\tau_{avg} = \frac{1}{t_f}\int_0^{t_f} \frac{kt}{kt_f}dt = \frac{1}{t_f}\int_0^{t_f} \frac{t}{t_f}dt$$

$$= \frac{(t^2)_0^{t_f}}{t_f^2} = \frac{1}{2} - 0 = \frac{1}{2}$$

When the conversion of ethyl ligand is a 50%

$$x(t) = \frac{1}{2} = \frac{kt}{kt_f}$$

$$\frac{t_f}{2} = t$$

$$\frac{29.7 \pm 6.31}{2}\text{min} = 14.9 \pm 3.16\, min$$

An additional experiment was performed at 303 K and had an average time scale of 27.0 ± 3.18 min, which equates to a single turnover of 0.556 ± 0.0654 h^{-1} per Ir atom. The data for the timescale as well as formation of ethyl bands are shown below. The two data sets provide an activation energy of 62 kJ/mol, a near match to the steady state activation energy reported in Table S3 of 64 kJ/mol.

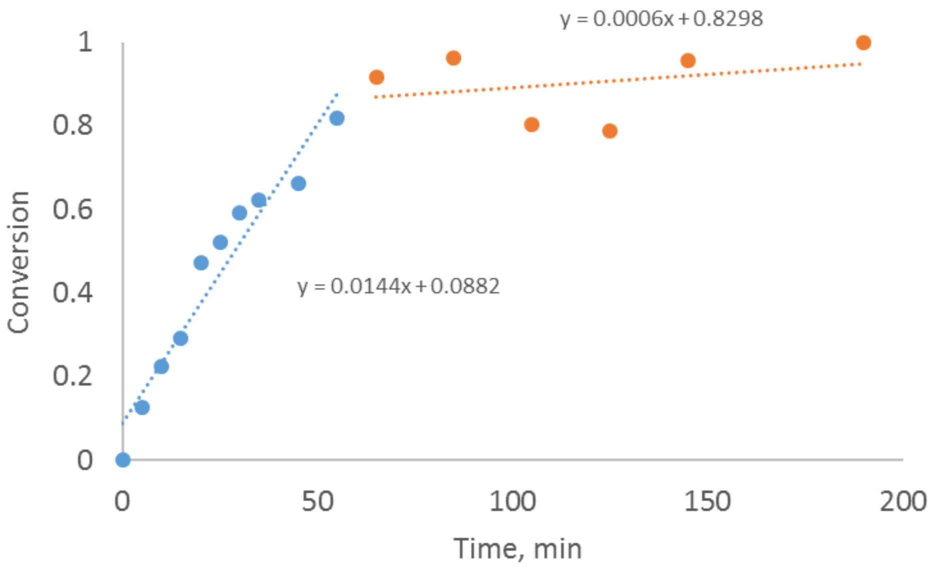

Figure S25. FTIR data of the removal of ethyl ligand at the ν_{CH} region, where intensity is plotted as conversion assuming all of the ethyl ligand has been removed at steady state. Flow conditions were: 1 bar, 303 K, He = 10 mL/min, H_2 = 10 mL/min.

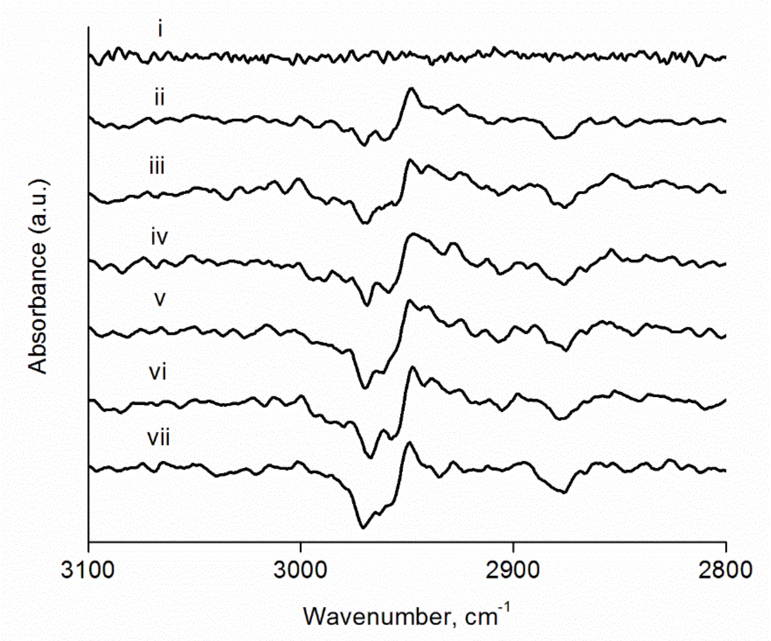

Figure S26. Difference spectra in ν_{CH} region of silica-supported samples formed from **1** by reactive decarbonylation with TMAO in the presence of ethylene at . Data represent changes occurring in a flow system at 303 K under the following conditions: (i) helium flowing at a rate of 10 mL/min and subsequently H_2 flowing at a rate of 10 mL/min of for the following times (min): (ii) 10, (iii) 20, (iv) 45, (v) 55, (vi) 65, (vii) 120.

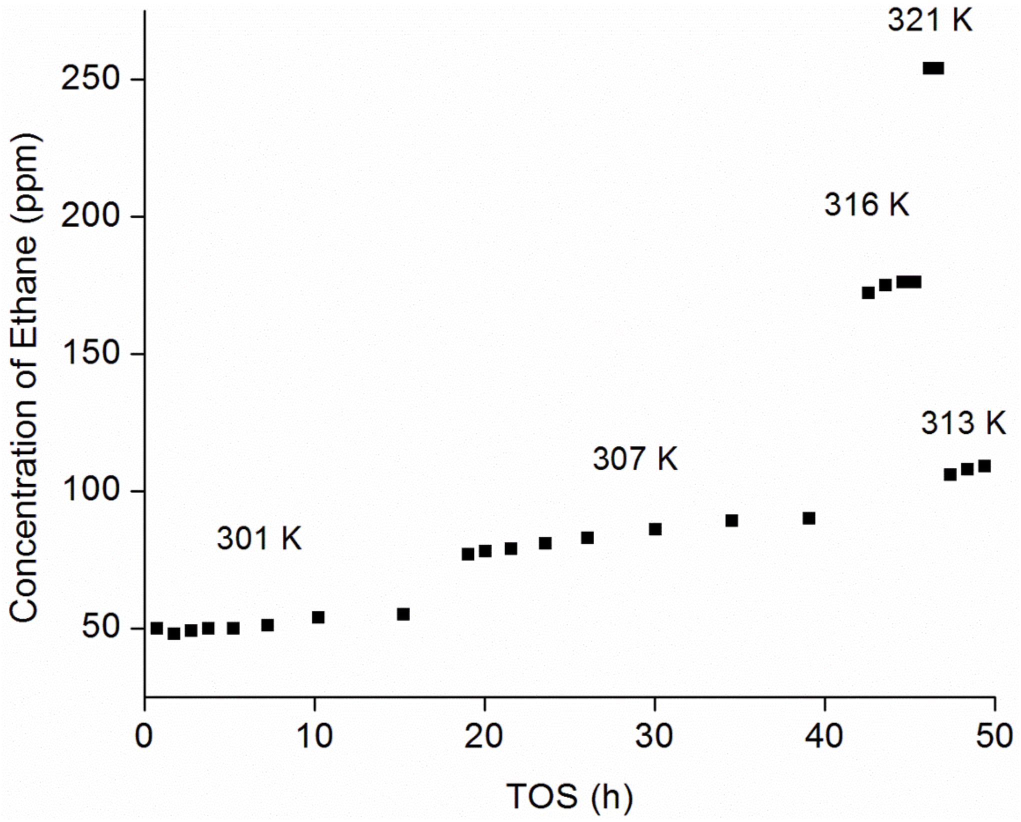

Figure S27. Steady-state ethylene hydrogenation at variable temperatures. The total flow rate was 63 mL/min, 1 bar (10 mL/min H_2, 3 mL/min C_2H_4, and a balance of helium).

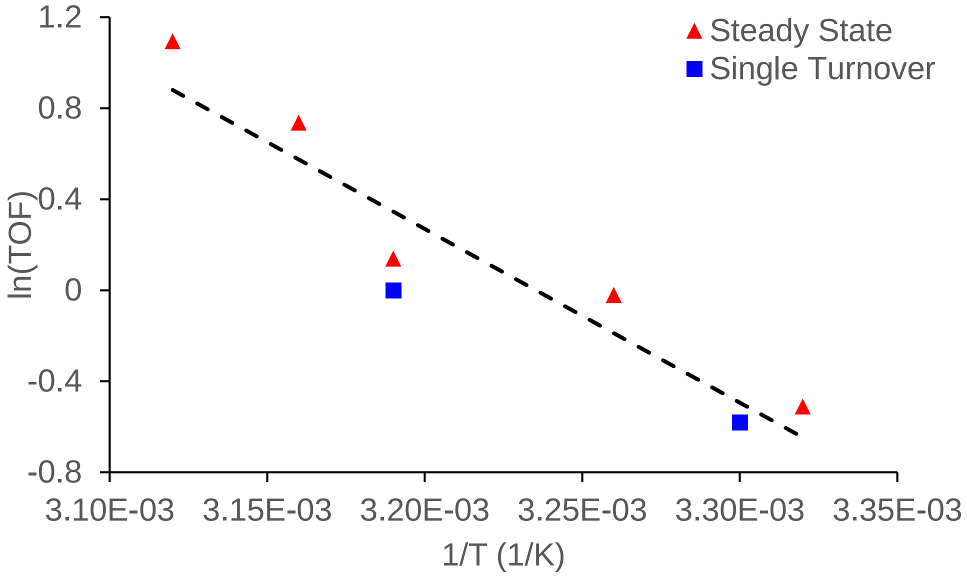

Figure S28. Arrhenius plot representing the same apparent activation energy (as represented by the slope) based on data from single-turnover (followed by FTIR spectroscopy in Figures 2 and S25) and steady-state ethylene hydrogenation experiments of Figure S26.

Experimental

Materials and sample preparation

Detailed synthesis in preparation of **1** and non-imprinted clusters are reported elsewhere.[S5] Imprinted clusters were made using the same method but contained an ethylene atmosphere (Praxair, 99.999%) during TMAO treatment. All clusters were supported on dehydroxylated silica (Degusa Aerosil 200) at 723 K by a *n*-hexane (EMD Chemicals, 95%) slurry. Silica was initially hydroxylated by refluxing deionized water overnight. The silica-water mixture was cooled to room temperature and was centrifuged (Thermofisher, Sorvall RC6) to separate the solid phase from the supernatant. The silica was then dried under vacuum at 473 K for 15 h and was ground into a fine powder. Calcination condition occurred at 723 K (temperature ramp, 5 K / min) under dry air for 4 h followed by 10 hours of helium flow. BET surface are of the calcined silica was 190 +/- 3 m^2/g.

n-heaxane was distilled and dried (sodium hydride) prior to dissolving the iridium clusters. The clusters were supported in a slurry of *n*-hexane using Schlenk line techniques. Upon contact with support the solution quickly changed colors from yellow to colorless indicating that the clusters were successfully transferred from solution to the silica. The solution underwent 1 hour of stirring followed by a 24 h evacuation (15 mtorr) at room temperature. Each sample contained a 1 wt% Ir loading. Samples were stored in an argon glovebox (O_2 and H_2O monitors reading < 5 ppm). All samples were handled using air exclusion techniques.

Silica was initially hydroxylated by refluxing in deionized water overnight; the silica-water mixture was cooled to room temperature and centrifuged to remove the supernatant liquid. The silica was dried under vacuum at 473 K for 15 h and ground into a fine powder, then calcined at 773 K in dry air for 4 h followed by 10 h in flowing helium. The BET surface area of the calcined silica was 190 ± 3 m2/g.

Analysis of Liquid Samples

All samples were prepared with a distilled decane solvent (>99% Sigma-Aldrich) because of its low volatility. Liquid samples were characterized by infrared spectroscopy with a Bruker Tensor 27 instrument, spectral resolution 2 cm^{-1}, using an IR cell (CaF_2 windows, d = 0.5 mm).

In-situ Fourier-Transform Infrared (FTIR) Spectroscopy and Mass Spectrometry

A Bruker IFS 66v/S spectrometer with a spectral resolution of 2 cm^{-1} was used to collect IR difference spectra and a Nicolet 6700 FTIR spectrometer with a spectral resolution of 4 cm^{-1} was used to collect transmission infrared spectra of the supported catalyst samples. Approximately 30 mg of sample, 1wt% of Ir, was pressed into a wafer in an argon glovebox (MBraun, H_2O < 0.5 ppm and O_2 < 5 ppm) and loaded into an in-situ cell that serves as a flow reactor (In-situ Research institute, Inc., South Bend, IN). The cell was sealed and promptly connected to a flow system to prevent air exposure. All lines were purged with helium (Airgas, 99.999%) prior to connecting the cell. Only hydrogen (Airgas 99.999%) was used as a reactive gas for in-situ FTIR

characterization. Gasses were purified by passing through Cu/Al_2O_3 and activated zeolite 4A to remove traces of O_2, moisture, and hydrocarbons.

The mass spectrometer was placed in tandem with the FTIR spectrometer. Mass spectra of effluent gas from the in-situ cell were measured by an online Balzers OmniStar mass spectrometer running in multi-ion monitoring mode. Major fragments of ethane were monitored during hydrogenation, C_2H_6 (*m/z* = 26, 27, 28, 29, 30).

Catalytic Performance

Ethylene hydrogenation was carried through a once-through packed-bed flow reactor under differential conditions. 250 mg of catalyst (1.0 wt% Ir) was loaded into a U-shaped reactor in an argon-filled glovebox and immediately installed in the flow system of avoid air exposure. Process lines of the flow unit and reactor are purged with helium (Praxair, 99.999%). Reaction conditions occur at 40° C and at atmospheric pressure. Temperature is monitored by a thermocouple placed directly upstream of the catalyst bed. The total flow rate during catalysis was 63 mL/min (10 mL/min H_2 (Praxair, 99.999%), 3 mL/min C_2H_4 (Praxair, 99.999%), and a balance of helium). All gasses were purified through traps to remove traces of O_2, moisture, and hydrocarbon prior to the catalyst bed. Ethylene and ethane concentrations were monitored by an online MKS FTIR gas analyzer (Multigas 2030); ethane was confirmed to be the only product via online gas chromatography.

NMR Spectroscopy and Resonances

The propoxy-alkyl substituents of the cluster-bound calixarene-phosphine macrocycles consist of two sets of carbons belong to axial and equatorial calixarene ligands, in the expected 1 (axial):2 (equatorial) ratio. The axial calixarene alkyl carbons of the lower-rim substituents are shifted to lower field versus the equatorial ones. Propoxy carbons of the axialcalixarene ligand give two resonances in a 2:1 ratio. In contrast, the pattern of equatorial calixarene propoxy carbons is more complex, as a result of magnetically non-equivalent environment for each propoxy tether, and therefore consists of three resonances in a 1:1:1 ratio. Methylene carbons directly substituted at P resonate at 78.61 ppm and are present as a doublet of doublets, which originates from the coupling of phosphorus and hydrogens with $^1J_{CP}$ = 12.87 Hz, $^1J_{CH}$ = 6.44 Hz. Bridging methylene carbons of the axial and equatorial calixarene skeleton overlap, and give seven resonances at 33.34 ppm, 33.41 ppm, 33.56 ppm, 33.59 ppm, 34.03 ppm, 34.05 ppm, and 34.06 ppm. The same number of resonances arises from aromatic carbons that are directly connected to oxygen, which is in agreement with the cluster geometry proven by single-crystal X-ray diffraction. The mostlow field resonances at 214.21 ppm and 218.56 ppm are assigned to bridging carbonyls, as a result of greater deshielding of those carbons due to donation of electronic density to two iridium atoms in the bridging position, versus a single iridium atom for terminal carbonyls. The ratios of carbonyls are 2:1 in agreement with the observed equatorial:axial substitution pattern in the cluster (i.e. 2 equatorial and a single axial bound calixarene-phosphine ligand). The assignment of all other carbonyls was made on the basis of carbon-phosphorus coupling. The largest $^3J_{CP}$ = 24.1 Hz is characteristic to coupling of ligands in the trans position. In the cluster, this trans coupling can be uniquely assigned to a single carbonyl in the apical position, which is coupled to the P of the axial-phosphine ligand. The remaining two apical carbonyls yield a sharp resonance at 158.82 ppm. The

broadest carbonyl resonance at 175.19 can be attributed to the coupling of the radial CO to both axial and equatorial phosphorus, and yields a broad multiplet. The resonance at 159.80 is also a multiplet and can be attributed to the remaining 2 CO ligands in the basal plane, corresponding to axial carbonyl ligands.

Variable Temperature NMR was used to investigate the temperature dependence of NMR resonance width and chemical shift in temperature range 293K-313K. The chemical shifts in 13C NMR spectrum for all carbonyls does not change upon heating in this temperature range. Moreover, quantitative carbon NMR reflects a lack of any change in the integration of resonances (i.e. still produces a CO integration pattern of 2:1:1:1:2:2). This suggests a lack of exchange between different carbonyls in cluster at this temperature range.

Tetra-iridium-Tris(tert-butyl-calix[4]arene-tripropoxy-methyldiphenylphosphino)-cluster

^{1}H NMR ($CDCl_3$) δ 7.83-7.87 (br m, 8H, C_6H_5), 7.76, (m, 4H, C_6H_5), 7.39-7.44 (br m, 18H, C_6H_5), 7.13 (two d, 4H, ^{4}J= 1.5 Hz, ArH), 7.09 (d, 2H, ^{4}J= 1.5 Hz, ArH), 7.07 (d, 2H, ^{4}J= 1.5 Hz, ArH), 7.03 (d, 2H, ^{4}J= 1.5 Hz, ArH), 7.00 (d, 2H, ^{4}J= 1.5 Hz, ArH), 6.49 (s, 4H, ArH), 6.45 (s, 2H, ArH), 6.17 (d, 2H, ^{4}J= 1.5 Hz, ArH), 6.14 (d, 2H, ^{4}J= 1.5 Hz, ArH), 6.01 (s, 2H, ArH), 5.23 (m, 4H, CH_2P), 4.41 (d, 4H, ^{2}J= 12 Hz, $ArCH_2Ar$), 4.38 (m, 2H, CH_2P), 4.34 (d, 2H, ^{2}J= 12 Hz, $ArCH_2Ar$), 4.06 (d, 2H, ^{2}J= 12 Hz, $ArCH_2Ar$), 3.96 (d, 2H, ^{2}J= 12 Hz, $ArCH_2Ar$), 3.79-3.86 (br m, 9H, OCH_2), 3.67-3.72 (br m, 3H, OCH_2), 3.64 (t, 4H, ^{3}J= 6 Hz, OCH_2), 3.58 (t, 2H, ^{3}J= 6 Hz, OCH_2), 3.12 (d, 4H, ^{2}J= 12 Hz, $ArCH_2Ar$), 3.07 (d, 2H, ^{2}J= 12 Hz, $ArCH_2Ar$), 2.85 (d, 2H, ^{2}J= 12 Hz, $ArCH_2Ar$), 2.70 (d, 2H, ^{2}J= 12 Hz, $ArCH_2Ar$), 2.68 (d, 2H, ^{2}J= 12 Hz, $ArCH_2Ar$), 1.83-1.97 (br m, 18H, $OCH_2C\boldsymbol{H}_2$), 1.38, 1.40 (two s, 54H, *t*-Bu), 1.09 (t, 6H, ^{3}J= 6 Hz, CH_3), 1.04 (t, 3H, ^{3}J= 6 Hz, CH_3), 0.92 (m, 12H, CH_3), 0.89 (s, 18H, *t*-Bu), 0.87 (s, 9H, *t*-Bu), 0.79 (s, 18H, *t*-Bu), 0.77 (t, 6H, ^{3}J= 6 Hz, CH_3);

^{31}C qNMR ($CDCl_3$) δ : 218.56 (s, 2C, CO_{bridge}), 214.21 (s, 1C, CO_{bridge}), 175.19 (m, 1C, $CO_{terminal}$), 163.49 (d, $^{3}J_{CP}$ = 24.1 Hz, 1C, CO_{apical}), 159.80 (m, 2C, $CO_{terminal}$), 158.82 (m, 2C, CO_{apical}), 154.50 (m, 4C, $C_{ar}O$), 154.36 (s, 2C, $C_{ar}O$), 154.28 (br s, 2C, $C_{ar}O$), 153.67 (m, 1C, $C_{ar}O$), 152.58 (s, 2C, $C_{ar}O$), 152.48 (s, 1C, $C_{ar}O$), 144.39 (m, 6C, C_{ar}), 143.76 (m, 3C, C_{ar}), 143.35 (s, 1C, C_{ar}), 143.24 (s, 2C, C_{ar}), 135.68 (m, 6C, C_{ar}), 135.53 (m, 4C, C_{ar}), 135.37 (s, 2C, C_{ar}), 133.18 (m, 8C, C_{ar}), 132.54 (m, 4C, C_{ar}), 131.77 (m, 10C, C_{ar}), 131.56 (m, 1C, C_{ar}), 131.38 (m, 1C, C_{ar}), 130.26 (m, 6C, C_{ar}), 129.45 (s, 2C, C_{ar}), 129.30 (s, 2C, C_{ar}), 129.12 (s, 2C, C_{ar}), 128.28 (m, 6C, C_{ar}), 128.23 (m, 8C, C_{ar}), 125.77 (s, 6C, C_{ar}), 125.36 (m, 6C, C_{ar}), 124.50 (m, 6C, C_{ar}), 124.19 (m, 6C, C_{ar}), 78.61 (dd, 2C, $^{1}J_{CP}$ = 12.87 Hz, $^{1}J_{CH}$ = 6.44 Hz, $O\boldsymbol{C}H_2P$), 77.50 (br m, 4C, 1C+3C, $O\boldsymbol{C}H_2P$+$O\boldsymbol{C}H_2$),

76.12, 76.15 (two s, 2C+2C, O***C***H_2), 76.03 (s, 2C, O***C***H_2), 34.03, 34.04, 34.06 (three s, 2C+2C+2C, Ar***C***H_2Ar), 33.59 (s, 2C, Ar***C***H_2Ar), 33.56 (s, 1C, Ar***C***H_2Ar), 33.41 (s, 2C, Ar***C***H_2Ar), 33.34 (s, 1C, Ar***C***H_2Ar), 31.80 (s, 12C, *t*-***C***$_4H_9$), 31.77 (s, 4C, *t*-***C***$_4H_9$), 31.53 (br s, 2C, *t*-***C***$_4H_9$), 31.37 (br s, 2C, *t*-***C***$_4H_9$), 31.14, 31.17, 31.18 (three s, 8C+8C+4C, *t*-***C***$_4H_9$), 31.11 (s, 4C, *t*-***C***$_4H_9$), 31.08 (br s, 4C, *t*-***C***$_4H_9$), 23.52 (s, 2C, OCH_2***C***H_2CH_3), 23.46 (s, 1C, CH_2***C***H_2CH_3), 23.21 (s, 2C, CH_2***C***H_2CH_3), 23.16 (s, 2C, CH_2***C***H_2CH_3), 23.03 (s, 2C, CH_2***C***H_2CH_3), 10.75 (s, 2C, CH_2***C***H_3), 10.70 (s, 1C, CH_2***C***H_3), 10.16, 10.19 (two s, 2C+2C, CH_2***C***H_3), 9.87 (s, 2C, CH_2***C***H_3).

^{31}P NMR ($CDCl_3$) δ 18.41, -11.35.

References

S1. A. M. Argo, J. F. Odzak, F. S. Lai, B. C. Gates, *Nature* **2002,** *415,* 623-626

S2. M. K. Ko, H. Frei, *J. Phys. Chem. B*, **2004,** 108, 1805-1808

S3. C. Preti, G. Tosi, *Transition Met. Chem.* **1976,** 2, 1

S4. A. Peremans, A. Tadjeddine, *J. Chem. Phys.* **1995,** 103, 7197-7203

S5. A. Orkut, R. C. Runnebaum, X. Ouyang, J. Lu, C. Aydin, S. Hwang, S. Zhang, O. A. Olatunji-Ojo, K. A. Durkin, D. A. Dixon, B. C. Gates, A. Katz, *Nat. Nanotechnol.* **2014,** *9,* 459-465
